# Slow Magnetic Relaxation of Dy Adatoms with In-Plane Magnetic Anisotropy on a Two-Dimensional Electron Gas

**DOI:** 10.1021/acsnano.2c04048

**Published:** 2022-06-30

**Authors:** Valerio Bellini, Stefano Rusponi, Jindřich Kolorenč, Sanjoy K. Mahatha, Miguel Angel Valbuena, Luca Persichetti, Marina Pivetta, Boris V. Sorokin, Darius Merk, Sébastien Reynaud, Dante Sblendorio, Sebastian Stepanow, Corneliu Nistor, Pierluigi Gargiani, Davide Betto, Aitor Mugarza, Pietro Gambardella, Harald Brune, Carlo Carbone, Alessandro Barla

**Affiliations:** 1 S3-Istituto di Nanoscienze-CNR, Via Campi 213/A, I-41125 Modena, Italy; 2 Institute of Physics, Ecole Polytechnique Fédérale de Lausanne (EPFL), Station 3, CH-1015 Lausanne, Switzerland; 3 Institute of Physics (FZU), Czech Academy of Sciences, Na Slovance 2, CZ-182 21 Prague, Czech Republic; 4 Istituto di Struttura della Materia (ISM), Consiglio Nazionale delle Ricerche (CNR), I-34149 Trieste, Italy; 5 School of Physics and Materials Science, Thapar Institute of Engineering and Technology, Patiala 147004, India; 6 Catalan Institute of Nanoscience and Nanotechnology (ICN2), CSIC and BIST, Campus UAB, Bellaterra, E-08193 Barcelona, Spain; 7 Instituto Madrileño de Estudios Avanzados en Nanociencia (IMDEA Nanoscience), E-28049 Madrid, Spain; 8 Department of Materials, ETH Zurich, CH-8093 Zurich, Switzerland; 9 Dipartimento di Fisica, Università di Roma “Tor Vergata”, I-00133 Roma, Italy; 10 ALBA Synchrotron Light Source, E-08290 Cerdanyola del Vallès, Spain; 11 European Synchrotron Radiation Facility, F-38043 Grenoble Cedex, France; 12 Institució Catalana de Recerca i Estudis Avançats (ICREA), Barcelona E-08010, Spain

**Keywords:** slow magnetic relaxation, single atom magnets, X-ray magnetic circular dichroism, density functional theory, perovskite oxides

## Abstract

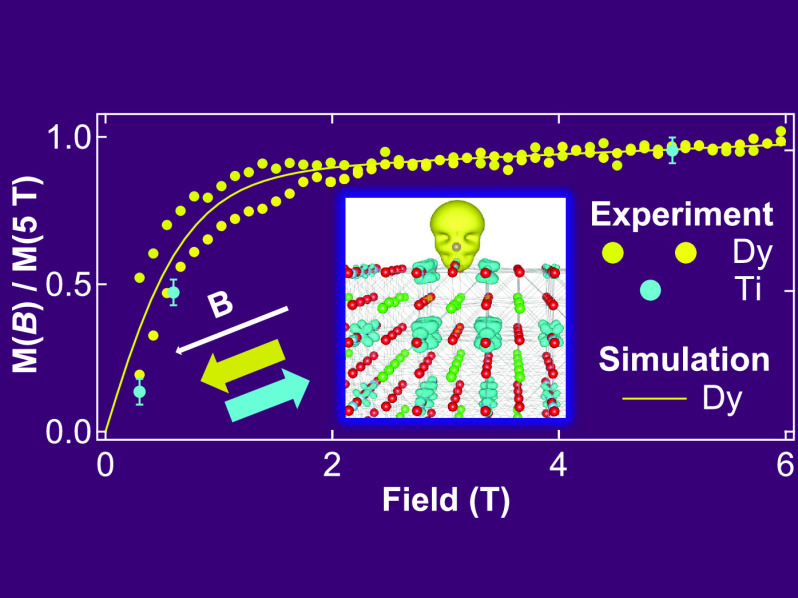

We report on the magnetic properties of Dy atoms adsorbed on the (001) surface of SrTiO_3_. X-ray magnetic circular dichroism reveals slow relaxation of the Dy magnetization on a time scale of about 800 s at 2.5 K, unusually associated with an easy-plane magnetic anisotropy. We attribute these properties to Dy atoms occupying hollow adsorption sites on the TiO_2_-terminated surface. Conversely, Ho atoms adsorbed on the same surface show paramagnetic behavior down to 2.5 K. With the help of atomic multiplet simulations and first-principles calculations, we establish that Dy populates also the top-O and bridge sites on the coexisting SrO-terminated surface. A simple magnetization relaxation model predicts these two sites to have an even longer magnetization lifetime than the hollow site. Moreover, the adsorption of Dy on the insulating SrTiO_3_ crystal leads, regardless of the surface termination, to the formation of a spin-polarized two-dimensional electron gas of Ti 3d_*xy*_ character, together with an antiferromagnetic Dy–Ti coupling. Our findings support the feasibility of tuning the magnetic properties of the rare-earth atoms by acting on the substrate electronic gas with electric fields.

The study of the interaction between a magnetic impurity and a nonmagnetic host is of fundamental interest, as the hybridization between the two determines the electronic and magnetic properties of the system, including its anisotropy. The magnetism of transition-metal atoms on surfaces has been an active field of research for almost 20 years,^1−9^ while investigations of the magnetic properties of rare-earth (RE) individual atoms are more recent.^10−22^ The latter studies have recently led to the discovery of single atom magnets (SAMs), based on either Ho or Dy atoms deposited on MgO/Ag(100)^21,22^ or Dy atoms on a graphene/Ir(111) substrate.^14^ These systems confirmed previous expectations that long magnetic relaxation times could be reached with strongly axial chemical bonds leading to uniaxial magnetic anisotropy.^21,23^ In the case of Ho/MgO/Ag(100), it was found that a single Ho atom is able to keep its magnetization for at least tens of minutes at temperatures up to 35 K.^21,24,25^ For Dy/MgO/Ag(100), the stability of the magnetization at *T* ≤ 15 K extends to several days.^22^ This extraordinary stability, like in the case of lanthanide-based single-molecule magnets,^26−28^ results from a symmetry-protected magnetic ground state, achieved through a strongly axial crystal field interaction, and the decoupling of the RE spin from the underlying metal through the MgO layers, preventing spin reversal due to scattering with electrons and phonons.^21^ In comparison, under identical adsorption conditions on a MgO layer, transition-metal single atoms show a large magnetic anisotropy but magnetization lifetime in the range of milliseconds.^5,6,29^

In Ho/MgO/Ag(100), direct manipulation of the spin of the Ho atoms (i.e., read and write) with a scanning tunneling microscope (STM) tip was demonstrated,^24,30,31^ highlighting the potential of these systems for information storage. Alternative routes to the control of the magnetic state of the lanthanide atoms may be achieved through their interaction with the substrate. These span from structural modifications that can lead to local variations of the crystal field potential impacting the charge and magnetic anisotropy of the rare-earth atoms, to electronic modifications, such as variations of the surface electron density influencing the spin reversal rate of the lanthanide atoms.

A potential candidate as a support for rare-earth SAMs, allowing for the active control of both their structural and electronic properties, is the cubic perovskite dielectric oxide SrTiO_3_ (STO). This is a paradigmatic example of a quantum paraelectric material, where paraelectricity down to temperatures in the mK range is the result of the competition between ferroelectricity, quantum fluctuations, and structural distortions.^32−37^ Paraelectricity in STO is intimately coupled with the giant piezoelectric effect observed at cryogenic temperatures.^38,39^ These properties make STO a rich playground to study the effect of electric-field-induced changes of the local crystalline environment on the magnetic properties of the lanthanide atoms. Moreover, whereas bulk STO has a large band gap of 3.25 eV,^40^ its surface can host a high-mobility two-dimensional electron gas (2DEG).^41−45^ The density of carriers within this surface can be controlled either via the application of a gate voltage^46^ or through exposure to intense ultraviolet radiation.^43,47,48^ Both methods can be effective in controlling the scattering rate between the conduction electrons of the substrate and the localized magnetic moments of the lanthanide atom, thus offering a potential way to control the reversal of the rare-earth spins.

In this context, we have studied the structural, electronic, and magnetic properties of Dy atoms adsorbed onto STO(001) surfaces. We have found that Dy impurities are preferentially located at four-fold hollow sites of the TiO_2_-terminated surface, with a 4f^9^ configuration and a strong in-plane magnetic anisotropy. However, under the experimental conditions of this study, a significant minority of Dy adatoms adsorb at sites of the coexisting SrO termination. Dy atoms at the TiO_2_-terminated surface are found to be SAMs, characterized by an open magnetization cycle and spin relaxation times of the order of at least 800 s at 2.5 K. This was unexpected since this atomic species is characterized by an easy-plane magnetic anisotropy [i.e., there is no unique magnetic quantization axis in the STO(001) plane]. Finally, we find a significantly long-ranged antiferromagnetic coupling between Dy and Ti, related to the formation of a 2DEG at the STO surface upon Dy adsorption.

## Results and Discussion

**Figure 1:**
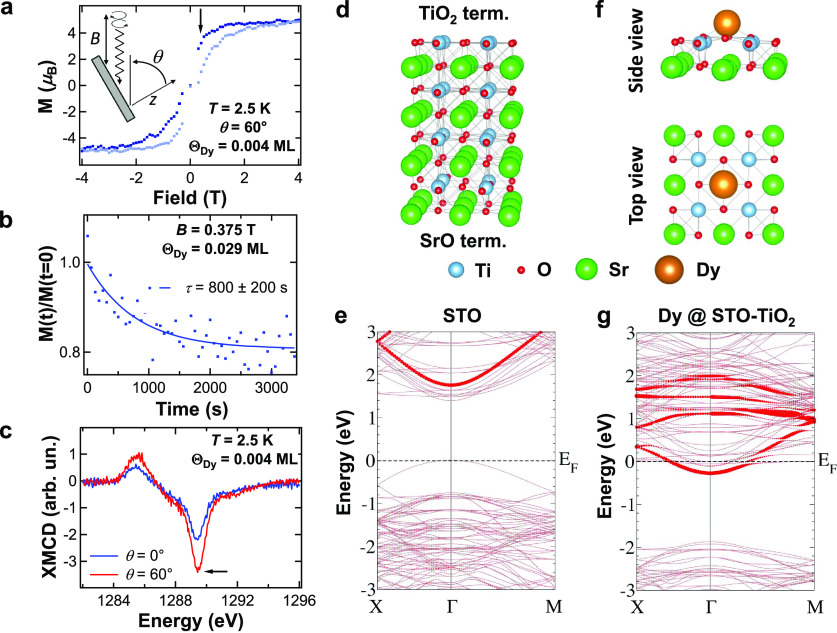
(a) Experimental magnetization cycle recorded by following the magnetic field dependence of the XMCD peak at the Dy *M*_5_ edge [see black arrow in panel (c)], at *T* = 2.5 K, d*B*/d*t* = 33.3 mT/s, θ = 60°, Θ_Dy_ = 0.004 ML (corresponding to a surface density of 0.026 Dy atoms/nm^2^). (b) Experimental magnetization relaxation curve (dots) and corresponding exponential fit (line), recorded at *B* = 0.375 T [see black arrow in panel (a)] after saturation at *B* = 5 T (Dy *M*_5_ edge, θ = 60°, *T* = 2.5 K, Θ_Dy_ = 0.029 ML). (c) Normalized XMCD spectra measured for magnetic field applied in the out-of-plane direction (θ = 0°) and predominantly in the STO(001) plane (θ = 60°), at the Dy *M*_5_ edge with *T* = 2.5 K, *B* = 5 T, Θ_Dy_ = 0.004 ML. (d,f) Sketches of the atomic structures and (e,g) corresponding electronic band structures for: (d,e) the plain STO substrate and (f,g) Dy adatoms (Θ_Dy_ = 0.25 ML) at the hollow site on the TiO_2_ terminated surface (spin up channel only). Although in the latter case only the top two STO atomic layers are visualized in the sketch for space reasons, the bands were calculated using the same 2 × 2 STO slab sketched in panel (d). In panels (e) and (g), the orbital projection on the Ti-d_*xy*_ character is highlighted, for the surface Ti layer only, by filled red circles whose size is proportional to its contribution at each eigenvalue. Note that the experimental data were acquired on Dy adsorbed on Nb:STO(001), while calculations are for a pure STO(001) cell.

The electronic and magnetic properties of Dy adatoms on the STO(001) surface were studied experimentally by polarization-dependent X-ray absorption spectroscopy (XAS) at the *M*_4,5_ edges, in particular by making use of X-ray magnetic circular dichroism (XMCD) and X-ray linear dichroism (XLD). The high sensitivity of these spatially averaging techniques allows measuring the properties of surface spins down to the noninteracting limit. Such a high magnetic dilution is achieved by depositing minute amounts of magnetic atoms at cryogenic temperatures, thus preventing their diffusion and consequent aggregation, and ensures that the magnetic properties are those of individual atoms, as demonstrated by comparison with single-atom scanning probe investigations on similar systems.^14,21,24,30,31,49^ The experimental investigations were complemented by atomic multiplet simulations and first-principles calculations based on the density functional theory (DFT) (see Methods for a description of experimental and theoretical techniques). As depicted in Figure 1a, Dy atoms adsorbed with very low density on the clean and ordered Nb:STO(001) surface (see Methods for the sample preparation procedure) show slow relaxation of the Dy magnetization, resulting in an open magnetization cycle at a temperature *T* = 2.5 K for magnetic fields up to *B* ≃ ±3 T. We observe a similar opening of the magnetization cycle in a wide range of Dy surface concentrations, up to Θ_Dy_ = 0.037 ML, and temperatures up to *T* = 6 K, while the opening is considerably reduced starting at Θ_Dy_ = 0.145 ML (see Supporting Information for the coverage dependence of the Dy magnetic properties). By following the decay of the XMCD amplitude as a function of time at a given magnetic field, after saturation at *B* = 5 T, we find the largest value of the magnetic lifetime τ of the Dy atoms at *B* = 0.375 T, where τ = 800 ± 200 s, as shown in Figure 1b. Moreover (see Figure 1c), dilute Dy atoms show an in-plane magnetic anisotropy, as indicated by the larger XMCD amplitude (relative to the XAS peak) at the *M*_5_ absorption edge when the magnetic field is applied close to the STO(001) plane (θ = 60°), as compared to the out-of-plane direction (θ = 0°). Very similar results are obtained for Dy adsorbed on the (001) surface of pure (i.e., without Nb doping) STO (see Supporting Information for a comparison between pure and Nb-doped STO), indicating that the extra conductivity achieved through Nb doping does not shorten the magnetization relaxation times. Dy/STO(001) can therefore be classified as a SAM, but unlike the previously reported cases of RE SAMs,^14,21,22,50^ all showing strong out-of-plane magnetic anisotropy, here the Dy atoms have in-plane magnetic anisotropy. Contrary to Dy, Ho single atoms show purely paramagnetic behavior down to *T* = 2.5 K, despite a magnetic anisotropy similar to that of Dy (see Supporting Information for the Ho/STO magnetic properties).

The recorded value of τ for Dy/STO(001) is comparable to that previously reported for Dy/graphene/Ir(111).^14^ Indeed, our DFT calculations indicate that the STO substrate, whose atomic structure is sketched in Figure 1d, shares some features with such graphene/metal substrates. The (001) surface of bare, stoichiometric STO is an insulator as depicted in Figure 1e. Oxygen atoms at the TiO_2_ terminated surface layer are responsible for the inverted parabolic band that reaches the Fermi level at the Γ point. On the other hand, it is well-known that oxygen vacancies act as charge donors and lead to the formation of a 2DEG at the STO surface.^42,43^ Here, we show that also the presence of Dy adatoms on the stoichiometric STO(001) surface, sketched in Figure 1f, leads to an electron doping, resulting in the partial filling of conduction bands. This effect is shown in Figure 1g, which depicts the spin up/majority channel bands (similar bands and projections are found in the spin down/minority channel) for a calculation based on a 2 × 2 cell, corresponding to a Dy concentration Θ_Dy_ = 0.25 ML. However, comparable results were obtained for a 4 × 4 cell (discussed later in Figure 6d), corresponding to Θ_Dy_ = 0.06 ML, very close to the coverage range used during the XMCD experiments. Moreover, although we depict here the case of the hollow adsorption site on the TiO_2_ termination (the different sites/terminations will be discussed at a later stage), the surface metallization occurs regardless of the crystal termination. It involves bands with predominant Ti 3d_*xy*_ character, related to surface Ti atoms in the case of a TiO_2_ termination, as shown in Figure 1g, and to subsurface Ti atoms in the case of a SrO-terminated crystal (see Supporting Information for the depth-dependence of this metallic state). Thus, our calculations show that, independently of the adsorption site, Dy deposition will lead to the formation of a 2DEG, reminiscent of what was previously observed at the oxygen-deficient STO surface.

**Figure 2:**
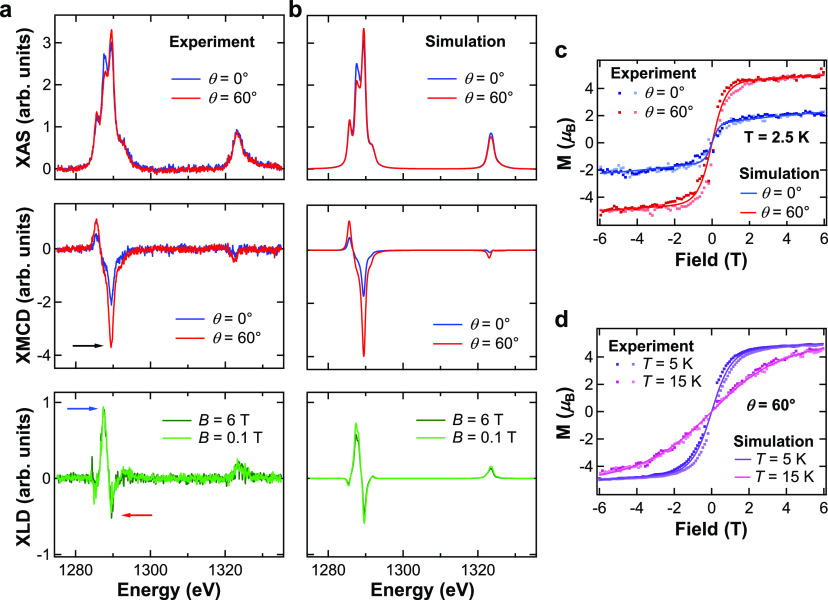
X-ray absorption spectra and magnetization curves of Dy atoms on a Nb:STO(001) surface (Θ_Dy_ = 0.037 ML). (a) XAS, XMCD, and XLD spectra measured at the Dy *M*_4,5_ edges and *T* = 2.5 K. The XAS and XMCD were recorded at *B* = 5 T, and the XLD was recorded at grazing incidence (θ = 60°). (b) XAS, XMCD, and XLD simulated by means of atomic multiplet calculations based on a point-charge model for the Dy crystal field, with the proportions between Dy species discussed in the text. (c) Experimental magnetization cycles (Dy *M*_5_ edge, *T* = 2.5 K, d*B*/d*t* = 33.3 mT/s) at normal (θ = 0°) and grazing (θ = 60°) incidence and corresponding simulated cycles at thermodynamical equilibrium based on the atomic multiplet model, with the proportions between Dy species discussed in the text. The experimental curves are normalized to the corresponding simulated curves at *B* = 6 T. Dark symbols are used for the downward magnetic field ramps (i.e., from positive to negative field), while light symbols are used for the upward field ramps. (d) Experimental magnetization cycles (θ = 60°, d*B*/d*t* = 33.3 mT/s) at various temperatures and corresponding simulated equilibrium curves based on the atomic multiplet model.

In order to rationalize our findings, we first analyze the XAS, XMCD, and XLD spectra of Dy adatoms deposited on the (001) surface of Nb:STO. Figure 2a displays the *M*_4,5_ XAS (top panel) characteristic for the Dy coverage range up to Θ_Dy_ = 0.037 ML. At the *M*_4,5_ edges, transitions from a 3d^10^ 4f^*n*^ state to a 3d^9^ 4f^*n*+1^ state are mainly excited, allowing one to probe the electronic and magnetic configuration of the rare-earth 4f shell. The spectral line shape of the XAS and XMCD (middle panel) is typical for a 4f shell occupation *n* = 9.^15^ Thus, adsorption at the STO(001) leads to a decrease of one electron in the occupation of this shell, characterized by *n* = 10 for Dy atoms in the gas phase. The in-plane magnetic anisotropy, related to the larger XMCD amplitude at grazing than at normal incidence, suggests that the ground state of individual Dy atoms on STO(001) is characterized by a low value of the projection of the total angular momentum *J* = 15/2 along the *z*-axis [corresponding to the (001) axis of the STO lattice]. The spectral shape of the XLD (bottom panel of Figure 2a) at the *M*_5_ edge is characterized by a large positive feature (blue arrow in the graph) followed, at higher energy, by a negative feature (red arrow). Such an XLD is characteristic for Dy when the 4f charge distribution is mostly pointing in the direction perpendicular to the STO surface.^51^ Due to the oblate character of the Dy(4f^9^) free-ion electron density, this charge distribution corresponds to a 4f magnetic moment pointing within the STO surface plane, consistently with the XMCD result.

**Figure 3:**
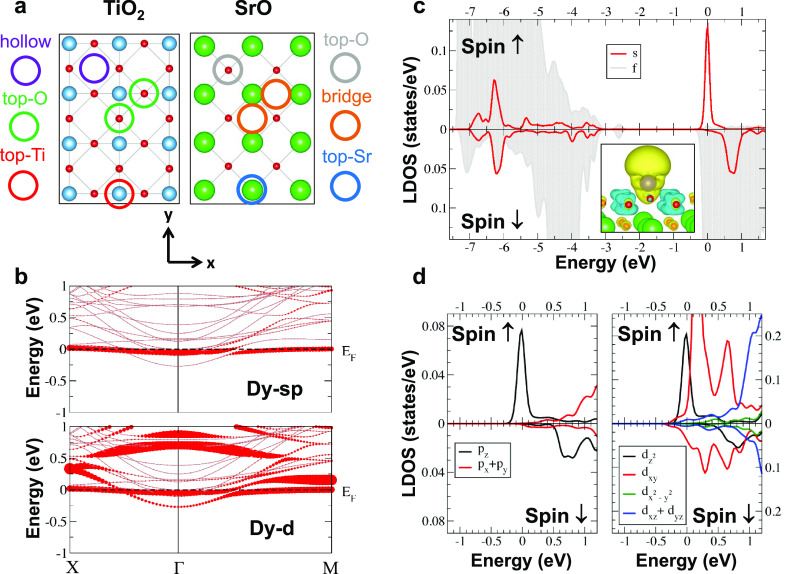
(a) Sketches of the six possible adsorption sites on TiO_2_ and SrO terminated STO(001) surfaces. The STO(001) single crystals used in the experiments are oriented as in the sketch, and both the magnetic field and the X-rays are always aligned within the *xz* plane. For the TiO_2_/top-O and SrO/bridge sites, with two-fold point symmetry, two inequivalent orientations are indicated. These are inequivalent with respect to the direction of magnetic field and X-rays. (b) Spin majority (↑) orbitally projected electronic band structure in the [−1 eV, +1 eV] energy range around the Fermi level for a Dy adatom at the TiO_2_/hollow adsorption site. The size of the red circles is proportional to the sp and d contribution to each eigenvalue. Spin-polarized LDOS projected on the (c) s and f and (d) p and d orbitals of a Dy adatom at the TiO_2_/hollow adsorption site. For p and d orbitals, the projection is carried out for each orbital symmetry separately. In the inset of panel (c), a close-up view of the spin-density isosurface at the Dy site is shown (isovalue of 10^–3^ e^–^/Å^3^). Yellow and cyan colors are associated with an excess of spin up and down electrons, respectively.

In view of the quantitative interpretation of the experimental XAS, XMCD and XLD, we have established by DFT the most stable adsorption configurations on both TiO_2_ and SrO crystal terminations and the corresponding occupation of 4f and valence (6s, 6p, and 5d) orbitals. Since our simulation cell (see Figure 1c) hosts both terminations simultaneously, we can compare the total energies of all six high-symmetry adsorption sites, sketched in Figure 3a. The total energies are tabulated in Table 1. The most stable adsorption configuration is found to be the hollow site of the TiO_2_ termination (Dy^*hollow*^ atoms), where Dy has a four-fold coordination to its O nearest neighbors. This is followed in energy by the top-O site at the SrO termination (Dy^*top*^ atoms), where Dy is axially coordinated to the underlying O atom, and its Sr next nearest neighbors lead to a four-fold symmetry. Since the method used for the preparation of clean STO(001) surfaces in vacuum is known to yield coexisting TiO_2_ and SrO terminations,^52^ it is instructive to analyze the relative stability of the different sites on the two terminations separately. Based on the results of Table 1, on the TiO_2_ surface, the energy differences between hollow and top-O/top-Ti sites are so big (more than 2.5 eV) that we expect the Dy adatoms to easily diffuse to the most stable four-fold hollow site, irrespective of where the atoms land at deposition. Therefore, a single site is expected on this termination. On SrO terraces, top-O and bridge sites are close in energy (the difference is only 0.6 eV), whereas the top-Sr site is significantly higher in energy (2.5 eV), compared to the top-O site. In such a case, there might be diffusion barriers between the top-O and the bridge sites, which cannot be overcome at our low deposition temperature, whereas diffusion from top-Sr to the other sites is likely to have no barrier. On this termination, we thus expect occupation of both top-O and bridge sites. On the bridge site, Dy has a two-fold coordination with its O nearest neighbors and Sr next nearest neighbors. In each SrO unit cell, there are two bridge sites with perpendicular projections in the *xy* plane of the O–Dy–O bond, as sketched in Figure 3a. This projection is aligned along *x* for one bridge site and along *y* for the other bridge site. In our experiments, the external magnetic field is always applied in the *xz* plane, thus making Dy atoms at the two bridge sites inequivalent. We thus label as Dy^*br*-O*x*^ Dy atoms at bridge sites with O–Dy–O bond projection along *x* and Dy^*br*-O*y*^ those at sites with O–Dy–O bond projection along *y*. Concerning the electronic configuration, Dy atoms at TiO_2_/hollow sites are found to have *n* = 9 electrons in the 4f shell. The same holds for the SrO/bridge site, while a small departure from the 4f^9^ configuration is observed for all other sites, except for TiO_2_/top-Ti, where the occupation is closer to 4f^10^. We find that for all adsorption sites the occupation of the valence orbitals of spd character is sizable. However, the magnetic polarization of each of these shells is very low, with spin moments below 0.1 μ_B_. Figure 3b shows the contributions to the band structure close to the Fermi level of majority spin sp and d Dy orbitals, while in Figure 3c,d, the spin-dependent local density of states (LDOS) is plotted for spdf Dy orbitals, for the case of the TiO_2_/hollow site (for p and d orbitals, the symmetry dependence is also given). The spatial localization of spd electrons on the Dy atoms is clearly evidenced by the nondispersive character of the partially occupied band close to *E*_F_. Indeed, inspection of the projected electronic density of states shown in Figure 3c,d reveals the presence of a localized band at the Fermi level, with high spin polarization and mixed 6s–6p_*z*_–5d_*z*^2^_ character. This can be nicely visualized in real space by plotting the spin-density isosurface around the Dy ion, as shown in the inset of Figure 3c. Its anisotropic shape with a vertically elongated spatial extension, going well beyond the Dy atomic position, cannot be accounted for only by the anisotropy of the well localized 4f shell. The larger spatial extension of 5d_*z*^2^_ and especially of 6p_*z*_ orbitals in the *z* direction, compared to that of the 4f shell, suggests assigning the spin cloud above the Dy atom to these orbitals. An anisotropic contribution from the 6s shell may also arise, due to the hybridization between this and the other valence orbitals.

**Table 1:** Total Energies (in eV) and Occupations (*n*) of the 4f Orbitals of the Six Stable Adsorption Sites for Dy on TiO_2_ and SrO Terminations^a^

Termination	Adsorption Site	Energy (eV)	*n* (e^–^)
TiO_2_	hollow	**+0.00**	9.00
	top-O	+2.61	9.39
	top-Ti	+3.22	9.71
SrO	top-O	+2.34 (**+0.00**)	9.38
	bridge	+2.95 (+0.61)	9.04
	top-Sr	+4.87 (+2.53)	9.37

a The values are relative to the energy of the TiO_2_/hollow site. In the case of the SrO termination, the energies in brackets are relative to the SrO/top-O site.

Having established the most likely occupied adsorption sites and the presence of a charge and spin cloud localized above the Dy atoms on hollow sites, we have simulated the XAS, XMCD, and XLD of Dy adatoms on the STO(001) surface by atomic multiplet calculations performed with the Quanty code^53^ (see Methods and Supporting Information for details about the atomic multiplet calculations). The system Hamiltonian includes Coulomb, spin–orbit, Zeeman, and crystal-field (CF) interactions acting on the 4f shell only. Figure 2b shows our best simulations, which reproduce very well the experimental data shown in the corresponding panels of Figure 2a. Our simulations, based on the combination of spectra characteristic for different adsorption sites, indicate that (66 ± 5)% of the Dy adatoms occupy Dy^*hollow*^ sites, (20 ± 2)% occupy bridge sites, equally distributed among Dy^*br*-O*x*^ and Dy^*br*-O*y*^ species, and (14 ± 2)% are in Dy^*top*^ sites. Thus, we confirm that TiO_2_ and SrO terminations coexist, with relative abundances of (66 ± 5)% and (34 ± 5)%, respectively. The relative abundances of Dy atoms at top-O and bridge sites on the SrO termination, (41 ± 6)% and (59 ± 6)%, respectively, suggest that atoms landing on top-Sr sites diffuse with approximately the same probability to either of those sites. Indeed, from the statistics of impact sites (there are two bridge sites, one top-O and one top-Sr site per SrO unit cell) and in the case of equal diffusion probability from top-Sr to top-O and bridge, we infer relative abundances for these sites of 37.5% and 62.5%, respectively, in agreement with the experimental values. The coexistence of Dy atoms at top-O and bridge sites of the SrO termination is similar to the case of rare-earth adatoms on the MgO surface (which is isostructural to the SrO termination discussed here), where adsorption at both top-O and bridge sites was experimentally observed by STM.^22,50,54^ The equilibrium magnetization cycles calculated by atomic multiplet calculations capture very well both the angular dependence of the experimental magnetization cycles, as shown in Figure 2c, and their temperature dependence up to *T* = 15 K, as depicted in Figure 2d. At 15 K, the cycle is closed, indicating that, at this temperature, magnetic relaxation is faster than the measurement time, which is of the order of 10 s.

**Figure 4:**
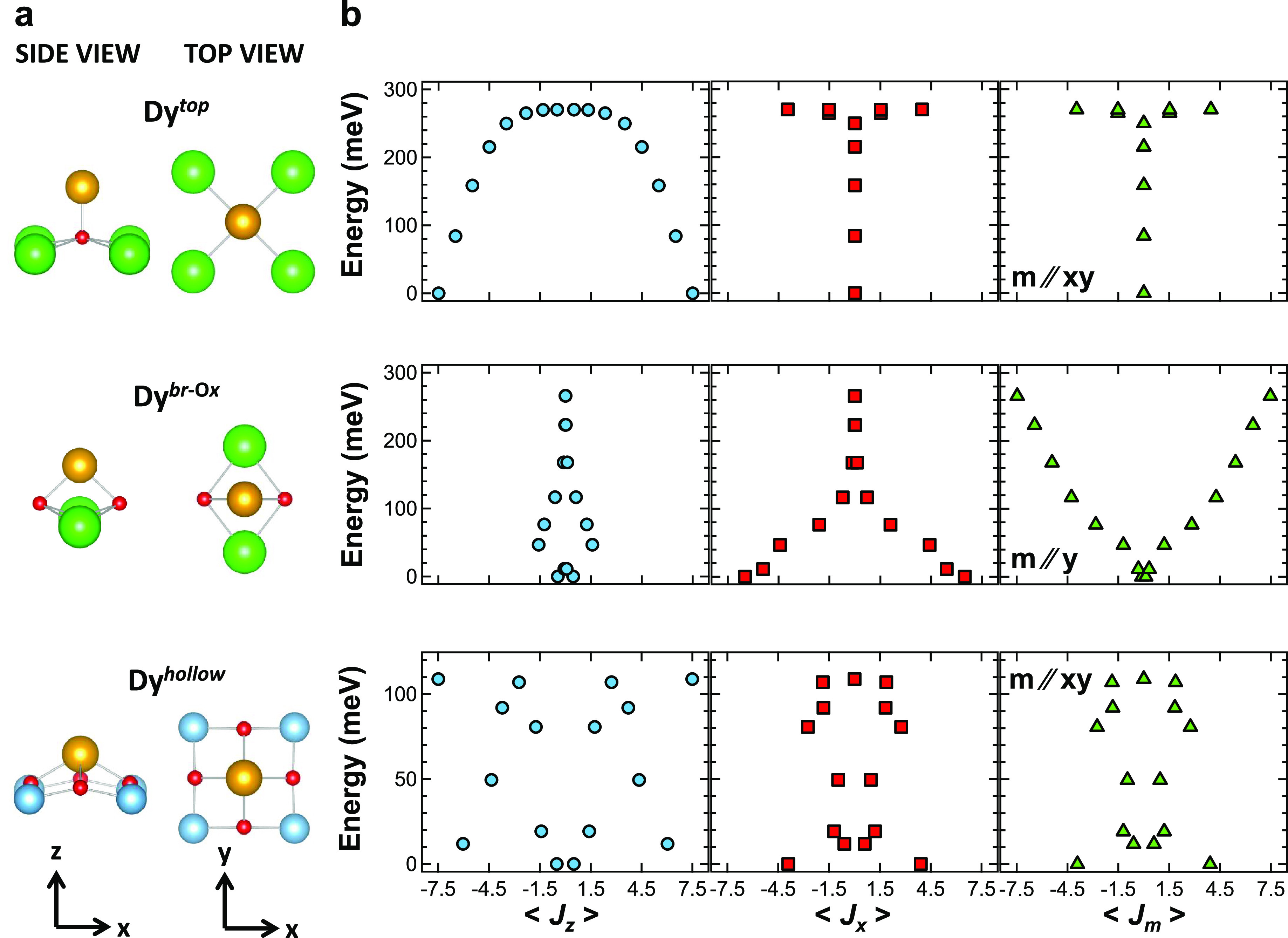
(a) Calculated adsorption configuration of the three sites at the origin of the simulated XAS, XMCD, and XLD spectra. The Dy atom and the first two coordination shells are shown. (b) Corresponding calculated energy scheme of the ground-state atomic *J* multiplet of Dy atoms, as obtained by diagonalization of the Hamiltonian defined for the atomic multiplet calculations, in a small magnetic field *B* = 1 μT applied along different high-symmetry directions (left column: *z*, middle column: *x*, right column: either *y* or *xy*). The small magnetic field is applied in order to define the quantization axis for each diagram.

Based on the atomic multiplet calculations, we can establish the 4f quantum level schemes and the ground-state wave functions for Dy in the three adsorption configurations used in our simulations, which are depicted in Figure 4a. Dy^*top*^ has a strong out-of-plane anisotropy, with a ground-state characterized by the maximum value of the total angular momentum *J* along the *z* direction, perpendicular to the STO(001) surface (see top panel of Figure 4b). The ground-state wave function is
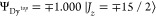
corresponding to a pure doublet, extremely well isolated from the first excited state (*ΔE* = 84.2 meV), and with a total barrier height of 270 meV. On the other hand, the two-fold *C*_2*v*_ symmetry of the bridge site leads to a strong in-plane uniaxial anisotropy. The maximum projection of *J* for Dy^*br*-O*x*^ atoms is along the *x* direction (see middle panel of Figure 4b), while it is along the *y* direction for Dy^*br*-O*y*^ atoms (not shown). Along the respective easy axes, the ground state wave function for these two species is
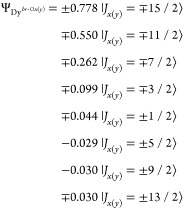
with a first excited state at *ΔE* = 11.3 meV and a total barrier height of 266 meV. Finally, on the hollow site of the TiO_2_ termination, the maximum projection of *J* lies within the STO(001) plane, independent of the direction (bottom panel of Figure 4b). This suggests an easy-plane type anisotropy, with the hard axis perpendicular to the STO surface. In the plane, the ground state of the Dy 4f^9^ configuration is
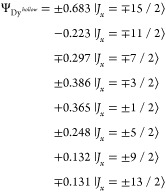
Even in this case, the ground doublet is well isolated from the first excited state (*ΔE* = 11.9 meV), while the total barrier height amounts to 109 meV.

**Figure 5:**
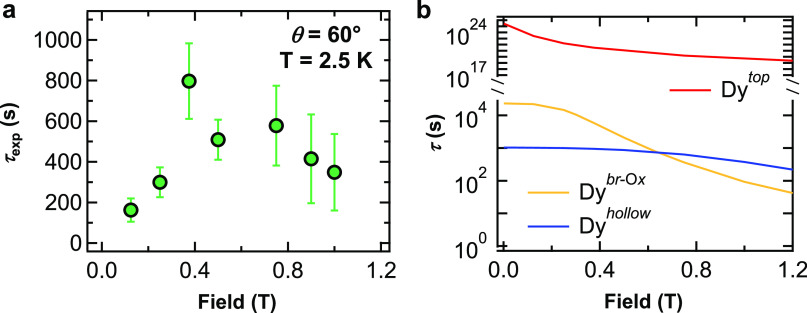
(a) Experimental magnetization relaxation time τ_exp_ of Dy atoms as a function of external magnetic field *B* (Θ_Dy_ = 0.029 ML, *T* = 2.5K, θ = 60°). (b) Intrinsic relaxation time τ calculated with the model described in the text for Dy^*hollow*^, Dy^*top*^, and Dy^*br*-O*x*^ atoms (*T* = 2.5K, θ = 60°).

Figure 5a shows the magnetic field dependence of the magnetization relaxation time τ_exp_ as recorded under low photon flux conditions (see Methods for the procedure used to measure τ_exp_ and for the definition of “low flux” conditions). As *B* is decreased from 1 to 0.375 T, τ_exp_ more than doubles, reaching a maximum value of 800 ± 200 s. At lower magnetic fields, however, τ_exp_ falls rapidly reaching a value of about 200 s at *B* = 125 mT. In order to determine which Dy species are responsible for the observed field dependence of τ_exp_, we note that, as shown in Figure 1b, at *B* = 0.375 T, the decrease of the magnetization over time from its initial to its equilibrium value is of the order of 20%. In our grazing incidence geometry, Dy^*hollow*^ atoms account for (69 ± 5)% of the total magnetization, while Dy^*br*-O*x*^ for (16 ± 2)%, Dy^*top*^ for (14 ± 2)%, and Dy^*br*-O*y*^ for only about 1%. Assuming that a Dy species retains its saturation magnetization, while the magnetic field is quickly ramped from 5 T down to 0.375 T without exposing the sample to the X-ray beam and based on the equilibrium magnetization curves calculated for each species (see Supporting Information), we expect a maximum decrease over time at 0.375 T of |*M*(0.375 T) – *M*(5 T)|/*M*(5 T) = 42% if the decay of *M* is due to Dy^*hollow*^ atoms, 8% in the case of Dy^*top*^, 6% for Dy^*br*-O*x*^, and about 1% in the case of Dy^*br*-O*y*^. In reality, due to its intrinsic finite lifetime, the magnetization partially relaxes already during the field ramp, so that the actual decrease of the magnetization over time at *B* = 0.375 T will be lower than the above estimate. We can thus conclude that the observed magnetization relaxation at θ = 60° is likely related to the Dy^*hollow*^ atoms.

Based on the magnetic field dependence of the electronic levels obtained by our multiplet simulations, we have calculated the magnetic field dependence of the intrinsic relaxation time τ with a spin–lattice relaxation model, including direct and Orbach-type scattering mechanisms, based on Fermi’s golden rule and the Hamiltonian proposed by Fort et al.,^55^ which involves transitions with *ΔJ*_*z*_ = ±1, ±2. A Debye model was used for the low-energy phonon spectrum. Spin-electron scattering was included based on the theory by Delgado and Fernández-Rossier,^56^ involving transitions between states with *ΔJ*_*z*_ = 0, ±1 (see Supporting Information for a detailed description of the magnetic relaxation model). We neglected the coupling with the spin of the Dy spd shells,^49^ due to their vanishing spin polarization. The spin–lattice and spin–electron scattering cross sections were adjusted so as to match the values of τ_exp_ in the magnetic field range 0.375 ≤ *B* ≤ 1 T, taking into account that τ_exp_ is related to τ by the expression τ_exp_^–1^ = τ^–1^ + τ_sec_^–1^, where τ_sec_ is a contribution to the experimental relaxation time arising from secondary electrons generated in the X-ray absorption process (see Supporting Information for an evaluation of τ_sec_). The calculation for Dy^*hollow*^, shown as a continuous blue line in Figure 5b, relates the decay of τ with increasing field beyond 0.3 T with an enhanced probability of spin–phonon scattering (see Figure S8a of the Supporting Information for the decomposition of τ into a spin–phonon and a spin–electron contribution). The field dependence of the lifetime for magnetic fields *B* < 0.3 T is not captured by our simplified model, which does not include Raman scattering mechanisms and local phonon modes. The drop of τ_exp_ at small fields may in fact originate in a two-phonon Raman mechanism similar to that found for Ho/MgO.^25^ We estimate that quantum tunneling due to the coupling of the 4f magnetic moment with the nuclear spin may be significant only for selected values of the magnetic field, in the range *B* ≲ 40 mT, thus it cannot explain the field dependence of τ in our investigated field range (see Figure S8b of the Supporting Information for the effect of the coupling between 4f moment and nuclear spin in the field range of the experiment). For comparison, assuming identical spin–lattice and spin–electron cross sections for all adsorption sites, we can estimate the magnetic field dependence of τ for Dy^*br*-O*x*^ and Dy^*top*^. These are also shown in Figure 5b. Dy^*top*^ shows values of τ which are orders of magnitude greater than those of Dy^*hollow*^. Indeed, the ground state of Dy^*top*^, an almost pure doublet with |*J*_*z*_| = 15/2, is expected to be particularly stable against spin–electron and spin–phonon scattering, even when projected at θ = 60°. In fact, the adsorption geometry of Dy^*top*^ is identical to that of both Dy and Ho on the top-O site of MgO. These show stable magnetization over time scales of hours or days (only limited by the time scales of the measurements), in a wide range of fields (up to at least 7 T for Dy and 8 T for Ho) and temperatures (up to at least 15 K for Dy and 40 K for Ho).^21,22,24^ Dy^*top*^ on SrO-terminated STO, together with its analogues on MgO, can be regarded as an approximate realization of a RE-O dimer, whose magnetization was predicted to be extremely stable by means of quantum chemistry calculations.^23,57^ We anticipate even longer intrinsic lifetimes for Dy^*top*^ when the magnetic field is applied perpendicular to the STO(001) surface, due to the strong out-of-plane anisotropy of this species. However, our magnetization cycles are recorded under X-ray flux conditions which severely limit the lifetime, especially at θ = 0°, where the density of the incident photon flux, twice as high as at θ = 60°, leads to a higher density of secondary electrons and thus a lower value of τ_sec_ by a factor of 4 (eq S7 of the Supporting Information). Under these conditions, we measure τ_exp_ = 160 ± 24 s at θ = 60° and we expect τ_exp_ ≲ 50 s at θ = 0°, fully limited by τ_sec_ (which is considerably lower than the value reported for Ho/MgO).^25^ Thus, we barely see an opening of the magnetization loop at normal incidence, as shown in Figure 2c. On the other hand, Dy^*br*-O*x*^ shows a 1 order of magnitude longer lifetime than Dy^*hollow*^ at low magnetic fields, but a much faster decay at high fields. Our qualitative comparison suggests that all considered adsorption sites of Dy on STO(001) have stable magnetization on time scales of at least a few hundreds of seconds. It is interesting that, although the Dy^*top*^ site with its out-of-plane magnetic anisotropy appears to be by far the most stable, the easy-plane configuration of Dy^*hollow*^ and the easy-axis in-plane configuration of Dy^*br*-O*x*^ may also lead to slow magnetic relaxation.

**Figure 6:**
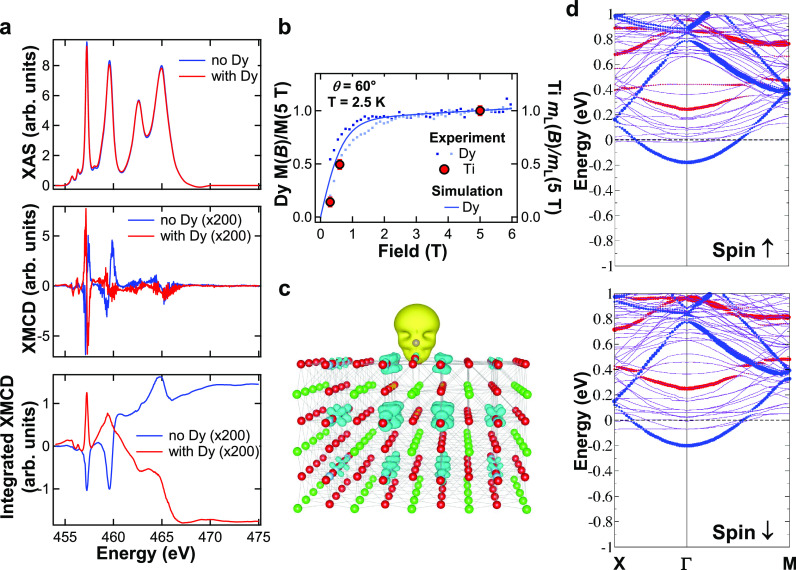
(a) Ti XAS (top panel) and XMCD (middle panel) spectra, with the corresponding XMCD integral (bottom panel) recorded at the *L*_2,3_ edges with *T* = 2.5 K, *B* = 5 T, and θ = 60°, before and after deposition of Dy (Θ_Dy_ = 0.035 ML) on the clean Nb:SrTiO_3_(001) surface. (b) Comparison between the magnetic field dependence of the Ti 3d orbital magnetic moment *m*_L_ (red dots) and the Dy total 4f moment (blue squares and line), both normalized at their corresponding values at *B* = 5 T. (c) Spin density isosurface of Dy/STO (Dy^*hollow*^ sites, 4 × 4 cell corresponding to Θ_Dy_ = 0.06 ML, GGA, isovalue of 10^–3^ e^–^/Å^3^), highlighting the magnetic polarization of Ti induced by Dy. Yellow and cyan colors are associated with an excess of spin up and down electrons, respectively. (d) Spin-resolved electronic band structures (top: spin-up, bottom: spin-down) for Dy adatoms (Θ_Dy_ = 0.06 ML) at the hollow site of the TiO_2_ terminated surface. The orbital projection on the Ti-d_*xy*_ character is highlighted by filled red circles (whose size is proportional to its contribution at each eigenvalue) for the surface Ti layer and by filled blue circles for the first subsurface Ti layer. The spin splitting at the Γ point amounts to 22 meV.

Stimulated by the finding of a Dy-adsorption-induced metallization of the STO substrate, we have investigated the influence of the Dy deposition on the magnetic properties of the STO surface. Figure 6a shows the XAS (top panel) and the corresponding XMCD (middle panel) recorded at the Ti *L*_2,3_ edges before and after deposition of 0.035 ML of Dy on Nb:STO(001). Prior to Dy deposition, the Ti XAS has the typical features of the 3d^0^ configuration.^58^ Moreover, Ti shows a small XMCD, with a positive integral (bottom panel). The XMCD amplitude at the most prominent peak of the *L*_3_ edge amounts to only about 0.6% of the corresponding edge jump. This is very similar to the XMCD previously found at the LaAlO_3_/SrTiO_3_ interface, on O_2_-annealed samples with a minimal amount of oxygen vacancies.^58^ Nominally Ti is in a tetravalent oxidation state, corresponding to a 3d^0^ configuration with no magnetic moment. However, covalence in the bond between Ti 3d and O 2p electrons leads to an actual 3d^(0+δ)^ occupation (where the value of δ depends on the degree of covalence), which can be associated with a small paramagnetic moment. A finite contribution to δ may also come from a small concentration of oxygen vacancies which may form at the surface during the sample preparation procedure or by irradiation with the X-rays. Although the magneto-optical sum rules^59,60^ cannot be applied to determine the spin magnetic moment at the Ti *L*_2,3_ edges, due to mixing of the *L*_2_ with the *L*_3_ intensity,^61^ we can extract an orbital magnetic moment *m*_L_ = −⟨*L*_*z*_⟩ = −0.003 ± 0.001 μ_B_ (assuming a vanishing δ and thus 10 holes in the 3d shell). Its negative sign suggests that, according to Hund’s rules, the Ti spin magnetic moment is aligned parallel to the applied magnetic field. The extremely small magnitude of the orbital moment supports that δ ≪ 1. After Dy deposition, the Ti XAS shows only minor variations, with a small decrease of the intensity of the sharp peaks and a consequent increase of the valleys between them. This indicates that, at least locally around the Dy impurities, the Ti 3d orbital occupation δ slightly increases, thus confirming that the Dy deposition actually dopes the STO(001) surface even in the case of Nb-doped STO crystals. The XMCD, on the other hand, shows no significant change of magnitude, but its sign and that of its integral are reversed. This implies that the Ti spin moment aligns antiparallel to the Dy spin moment, suggesting the onset of an antiferromagnetic coupling between the two. Indeed, Figure 6b shows that the magnitude of the Ti XMCD integral (proportional to the Ti orbital moment), normalized at its value at *B* = 5 T follows closely the normalized Dy magnetization curve. Thus, our experimental results are fully consistent with the first-principles calculated spin-density isosurface sketched in Figure 6c. Here, the opposite spin polarization of Ti with respect to Dy extends for several atomic distances, especially across Ti layers, and is maximal within the Ti subsurface layer, where Ti exhibits the highest spin magnetic moment *m*_S_ = −0.06 μ_B_/Ti atom, as compared to *m*_S_ = −0.03 μ_B_/Ti atom in the surface layer (note that these are the moments of the Ti atoms which, in each layer, are closest to the Dy atom). This finding correlates with the fact that the largest electron doping is found for the Ti-d_*xy*_ orbitals of the subsurface layer, whose bands are shown as blue circles in Figure 6d, while the Ti-d_*xy*_ bands of the surface layer (red circles) remain well above the Fermi level. We conclude that Dy induces a sizable spin polarization of the Ti atoms, which extends beyond the nearest-neighbor positions, suggesting that long-range Ti–Ti correlations may be active even at the extremely low Dy surface densities under study, likely due to the formation of the 2DEG within a couple of atomic layers at the Dy/STO interface.

## Conclusions

In conclusion, we have observed that Dy atoms adsorbed on the SrTiO_3_(001) surface show slow relaxation of the magnetization at temperatures *T* ≤ 6 K in an extended range of magnetic fields up to about 3 T. A careful comparison between experimental results, first-principles calculations, and a simple spin–lattice relaxation model allows us to attribute our observations to Dy atoms adsorbed at the hollow site of the TiO_2_ termination. These are characterized by an occupation of the 4f shell with 9 electrons and a strong easy-plane magnetic anisotropy, which results from the combined effect of the equatorial O and Ti ligands and a top charge due to strongly anisotropic, partially occupied Dy spd orbitals. The lifetime of the magnetic state, of the order of a few hundred seconds, is mainly limited by transitions between the two states of the ground-state doublet. In this geometry, Dy atoms induce a sizable spin polarization at the Ti atoms, whose magnetic moments couple antiferromagnetically with those of Dy. The formation of a spin-polarized 2DEG of Ti 3d_*xy*_ character at the Dy/STO interface offers promising ways for the electrical manipulation of the Dy magnetism. Besides tuning the density of carriers of the 2DEG, modifying the STO lattice through its piezoelectricity or exploiting the substrate magnetic moments induced by the fluctuating charge dipoles near the STO ferroelectric quantum critical point,^62^ one can envisage injecting/extracting a spin-polarized electrical current in/from the surface or subsurface TiO_2_ layer to “write/read” the Dy magnetization.

## Methods

Clean and ordered SrTiO_3_(001) surfaces were prepared by cycles of Ar^+^ sputtering and annealing in O_2_ atmosphere (partial pressure of *p* = 2–5 × 10^–6^mbar) at a temperature of 923 K on commercial SrTiO_3_ single crystals, either pure or doped with 1% at. Nb (the latter are referred to as Nb:STO in the manuscript). Nb:STO crystals were preferentially used, as their enhanced electrical conductivity compared to that of pure STO led to a higher signal-to-noise ratio of the XMCD measurements in the surface-sensitive total-electron-yield mode. The ordered surfaces exhibited sharp 1 × 1 LEED patterns (see Supporting Information), i.e., they were unreconstructed. Dy was evaporated, from thoroughly degassed rods or lumps in tungsten crucibles, onto SrTiO_3_(001) kept at *T* ≤ 6K, in order to prevent surface diffusion, and *p* ≤ 1 × 10^–10^mbar. The Dy coverage is expressed in monolayers (ML) relative to the SrTiO_3_(001) surface, where 1 ML is defined as 1 Dy atom per SrTiO_3_(001) unit cell (lattice parameter *a* = 0.3905 nm), corresponding to a surface density of 6.56 atoms/nm^2^. The coverage calibration based on the Dy XAS integral is obtained by comparison with previous investigations on Sm/graphene/Cu(111) (where STM and XAS were combined on the same sample) after proper rescaling of the different absorption coefficients and lattice parameters of the substrates and the number of holes of the rare-earth atoms.

The XMCD experiments were carried out at the EPFL/PSI X-Treme beamline^63^ of the Swiss Light Source (data taken at 2.5 and 15 K), at the BOREAS beamline^64^ of the ALBA synchrotron radiation facility (data taken at 5 K), and at the ID32 beamline^65^ of the European Synchrotron Radiation Facility (data not shown in the manuscript). The measurements were performed in the total-electron-yield mode at temperatures in the range 2.5–15 K, and magnetic fields up to 9 T, applied parallel to the X-ray beam. The energy resolution of the X-ray beam at the Dy *M*_4,5_ edges was of the order of at least 250 meV, the photon flux was of the order of 2 × 10^10^ photons/s, and both linear and circular X-rays were polarized to a degree close to 100%. The background-subtracted Dy *M*_4,5_ edge XAS [(*I*^+^ + *I*^–^)/2, where *I*^+^ and *I*^–^are the XAS spectra recorded with right and left circularly polarized X-rays, respectively], as shown in Figure 2a, is obtained by subtracting the X-ray absorption spectra of the bare SrTiO_3_(001) crystals, taken prior to Dy evaporation, from those of Dy/SrTiO_3_(001) recorded under identical conditions, and then subtracting step functions at the two edges. The XMCD is then calculated as *I*^–^ – *I*^+^. The XLD is defined as *I*^V^ – *I*^H^, where *I*^V^ and *I*^H^ are the XAS spectra recorded with vertically and horizontally linearly polarized X-rays. With reference to the sample orientation, as shown in Figures 3 and 4, the vertical linear polarization lies along the *y* axis, which corresponds to the sample rotation axis in our experiments, while the horizontal linear polarization lies within the *xz* plane. The magnetic field dependence of the magnetization relaxation time τ was recorded by saturating the Dy magnetization at a field of +5 T, then ramping the field to its target value at a speed of 33.3 mT/s and without X-rays on the sample, and finally recording the time dependence of the XMCD magnitude, which is fitted by a single exponential function.

DFT calculations were performed by means of the augmented-plane wave + local orbital method, as implemented in the Wien2K code,^66,67^ without including spin–orbit coupling. The in-plane STO lattice constant was fixed to the experimental value at *T* = 300 K, *a* = 0.3905 nm. The generalized-gradient approximation (GGA) of the exchange and correlation functional was considered for the structural characterization. Atomic relaxations of the coordinates of the Dy adatom and of the upper substrate layer atoms, carried out within the GGA functional,^68^ were allowed until residual forces were <1 meV/au. The electronic structure analysis reported in the main text was obtained by using an on-site version of the hybrid B3LYP functional,^69^ while, for testing purposes, an on-site Hubbard correction term (as implemented in the DFT+*U* method) on the f orbitals of Dy (*U* = 7 eV, *J* = 0.82 eV) was also considered. The former approach was found to describe the valence configuration of the Dy ions better, and a comparison between the two methods is given in the Supporting Information. Within the on-site B3LYP approach, a calculated electronic gap for STO bulk of 2.3 eV was found, to be compared with an experimental value of 3.25 eV.^40^ Further details on the simulation cell are given in the Supporting Information.

Atomic multiplet calculations were performed with the Quanty multielectron code,^53^ partially using the Crispy graphical interface,^70^ and used to simulate the temperature and magnetic field dependence of XAS, XMCD, XLD, and magnetization cycles. The Hamiltonian for the multiplet calculations includes electron–electron interactions, spin–orbit coupling, Zeeman energy due to the external magnetic field, and the crystal field potential acting on the Dy 4f shell. The electron–electron interactions (in terms of Slater–Condon integrals) as well as the spin–orbit coupling values were computed using Cowan’s atomic structure code. The Slater integrals were reduced to 66% of their atomic value in order to account for the screening due to surface electrons. The crystal field potential was calculated, for each adsorption site, by using an electrostatic point charge model, based on the optimized adsorption geometry and the Bader charges of the Dy neighbors, as obtained by our first-principles calculations (see Supporting Information for charge values and positions and the corresponding crystal field parameters in Wybourne notation). The final state Hamiltonian includes the presence of the core hole.

## Supporting Information Available

Complementary experimental data: STO(001) crystals characterization; Comparison between Nb-doped and pure SrTiO_3_; Coverage dependence of XAS, XMCD and magnetization cycles; XAS, XMCD, XLD and magnetization cycles of Ho/STO(001). DFT calculations: further details on the DFT simulation cell and the methodology; Comparison GGA+*U* vs on-site B3LYP; Spin and orbital projected band structures: 2DEG at the STO surface; Details about the atomic multiplet calculations: Point charges and crystal field parameters; Simulated XAS, XMCD, and magnetization cycles for each adsorption site; Details about the calculation of the magnetization relaxation time τ: theoretical model; Estimate of the secondary electrons contribution to the value of the relaxation time (PDF)
