# Supplementary material for: Slow Magnetic Relaxation of Dy Adatoms with In-Plane Magnetic Anisotropy on a Two-Dimensional Electron Gas
Source: ACS Nano. 2022 Jun 30;16(7):11182–93. doi: 10.1021/acsnano.2c04048 (PMC9330770; doi:10.1021/acsnano.2c04048)
Supplement: Supplementary file 1 — nn2c04048_si_001.pdf [file nn2c04048_si_001.pdf]

Supporting Information for: Slow magnetic relaxation of Dy adatoms with in-plane magnetic anisotropy on a two-dimensional electron gas

Valerio Bellini,[†] Stefano Rusponi,[‡] Jindřich Kolorenč,[¶] Sanjoy K. Mahatha,[§,‖] Miguel Angel Valbuena,[⊥,#] Luca Persichetti,[@,△] Marina Pivetta,[‡] Boris V. Sorokin,[‡] Darius Merk,[‡] Sébastien Reynaud,[‡] Dante Sblendorio,[‡] Sebastian Stepanow,[@] Corneliu Nistor,[@] Pierluigi Gargiani,[∇] Davide Betto,[††] Aitor Mugarza,[⊥,‡‡] Pietro Gambardella,[@] Harald Brune,[‡] Carlo Carbone,[§] and Alessandro Barla[*,§]

†*S3-Istituto di Nanoscienze-CNR, Via Campi 213/A, I-41125 Modena, Italy*

‡*Institute of Physics, EPFL, Station 3, CH-1015 Lausanne, Switzerland*

¶*Institute of Physics (FZU), Czech Academy of Sciences, Na Slovance 2, CZ-182 21 Prague, Czech Republic*

§*Istituto di Struttura della Materia (ISM), CNR, I-34149 Trieste, Italy*

‖*School of Physics and Materials Science, Thapar Institute of Engineering and Technology, Patiala - 147004, India*

⊥*Catalan Institute of Nanoscience and Nanotechnology (ICN2), CSIC and BIST, Campus UAB, Bellaterra, E-08193 Barcelona, Spain*

#*Instituto Madrileño de Estudios Avanzados en Nanociencia (IMDEA Nanoscience), E-28049 Madrid, Spain*

@*Department of Materials, ETH Zurich, CH-8093 Zurich, Switzerland*

△*Dipartimento di Fisica, Università di Roma "Tor Vergata", I-00133 Roma, Italy*

∇*ALBA Synchrotron Light Source, E-08290 Cerdanyola del Vallès, Spain*

††*European Synchrotron Radiation Facility, F-38043 Grenoble Cedex, France*

‡‡*Institució Catalana de Recerca i Estudis Avançats (ICREA), Barcelona E-08010, Spain*

E-mail: alessandro.barla@trieste.ism.cnr.it

Complementary experimental data

STO(001) crystals characterization

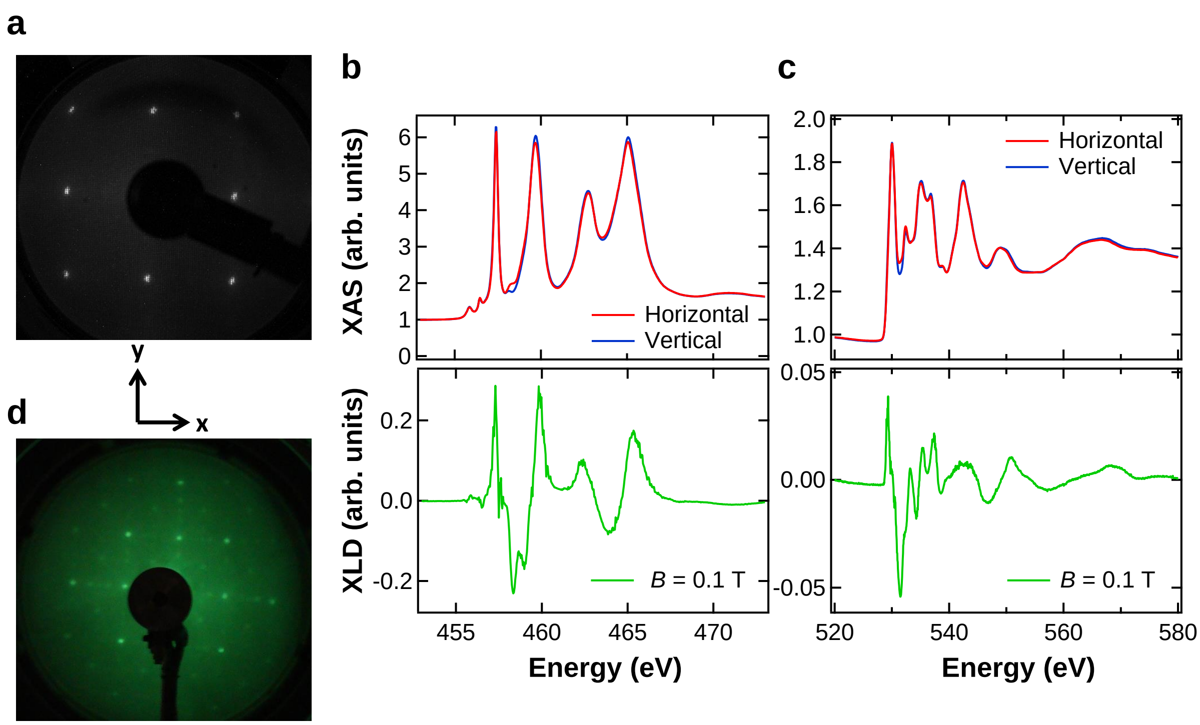

Figure S1: (a) LEED pattern of the clean and ordered Nb:SrTiO_3(001) surface taken at an electron beam energy of 90 eV. (b,c) XAS (top) and XLD (bottom) spectra of the STO(001) surface, measured at the (b) Ti $L_{2,3}$ edges and (c) O K edge, at a temperature $T = 2.5$ K and a magnetic field $B = 0.1$ T, at grazing incidence ($\theta = 60\,^\circ$). (d) LEED pattern of the reconstructed Nb:SrTiO_3(001) surface taken at an electron beam energy of 94 eV.

Fig. S1a shows the 1×1 Low Energy Electron Diffraction (LEED) pattern of the clean Nb:SrTiO_3(001) surface, following the preparation discussed in the Methods section. The sharp squared pattern, with the edges of the square aligned along the x and y axes, indicates that the surface is well ordered following the cubic structure of bulk STO, with no reconstruction. The crystals used in the XAS experiments were all oriented like in Fig. S1a, with x-ray beam and magnetic field aligned along the z axis (*i. e.* perpendicular to the plane of the figure) at normal incidence ($\theta = 0\,^\circ$), and within the xz plane at grazing incidence ($\theta = 60\,^\circ$). As shown in Figs. S1b and c, we observe sizable XLD both at the Ti $L_{2,3}$ and at

the O K edge, at $T = 2.5$ K, respectively. This arises from the anisotropy of the Ti 3d and of the O 2p orbitals within the almost cubic structure of STO at low temperatures.

An alternative preparation method was tested, which was reported to lead to a fully TiO_2-terminated surface.[1] Following this procedure, the as-received STO(001) crystals were first etched in a buffered solution of hydrofluoric acid (HF) for 30 s, then annealed in air at 1223 K for 1 hour. The samples were successively inserted in our UHV surface preparation chamber, where residual surface contamination was removed by annealing in O_2 atmosphere (partial pressure of $p = 2 \times 10^{-6}$ mbar) in two steps: first at 1023 K for 30 minutes and then at 1108 K for further 30 minutes. After cooldown to room temperature, LEED patterns were recorded at several electron beam energies, resulting in a reconstructed surface, with a coexistence of the previously reported p(2×1) and c(6×2) reconstructions,[2–4] as shown in Fig. S1d. As the coexistence of two reconstructions and the lack of knowledge of the precise atomic arrangement within the reconstructed surface would prevent us from determining the possible adsorption sites and the corresponding crystal field acting on the rare-earth adatoms, required to simulate the XAS, XMCD and XLD spectra, we did not investigate the magnetic properties of rare-earths on these samples.

Comparison between Nb-doped and pure $SrTiO_3$

Figure S2 shows a comparison between the XAS, XMCD, XLD and magnetization cycles of Dy adatoms adsorbed on Nb-doped (left column) and pure (right column) STO(001). Although the main spectral features are almost identical between the two cases, one observes a small reduction of the in-plane anisotropy for Dy on pure STO, with a corresponding increase of the XMCD at $\theta = 0^\circ$, and a reduction of the XLD amplitude. This is very likely due to a slightly different distribution of the Dy adatoms among the various adsorption sites, with a larger population of the top-O site of the SrO termination, characterized by an out-of-plane magnetic anisotropy, in the case of the pure STO(001) crystal. Despite these differences, the two magnetization cycles are almost identical, showing a clear hysteresis with

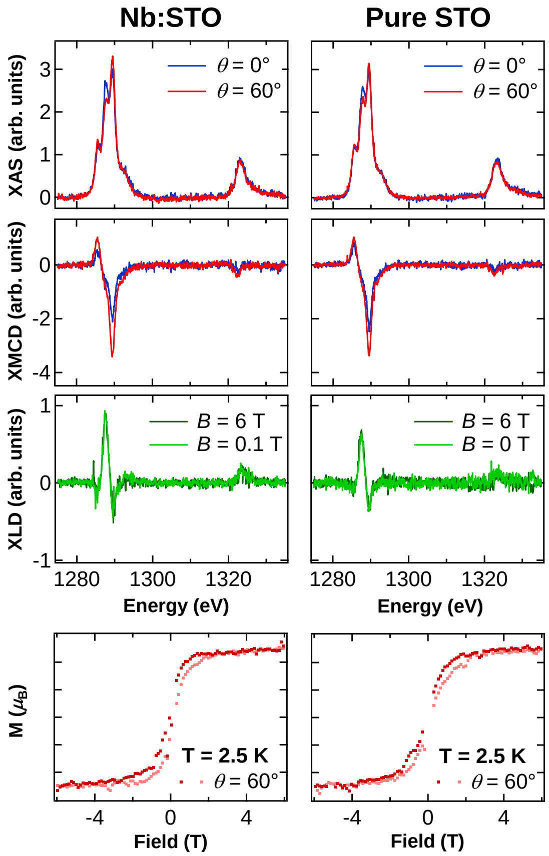

Figure S2: X-ray absorption spectra and magnetization curves of Dy adatoms on Nb:STO(001) (left column) and STO(001) (right column) surfaces ($\Theta_{\mathrm{Dy}} = 0.037\,\mathrm{ML}$, $T = 2.5\,\mathrm{K}$). The XAS, XMCD and XLD spectra were measured at the Dy $M_{4,5}$ edges; the XAS and XMCD were recorded at a magnetic field $B = 5\,\mathrm{T}$; the XLD was recorded at grazing incidence ($\theta = 60\,^{\circ}$). The magnetization curves were recorded at $\mathrm{d}B/\mathrm{d}t = 33.3\,\mathrm{mT/s}$ and grazing ($\theta = 60\,^{\circ}$) incidence. Dark symbols are used for the downward magnetic field ramps (*i. e.* from positive to negative field), while light symbols are used for the upward field ramps.

approximately the same area in both cases. This indicates that, under the same experimental conditions, Dy has nearly identical magnetization relaxation time on both single crystals, thus ruling out any significant role of the Nb doping in the magnetic stability of the Dy adatoms.

Coverage dependence of XAS, XMCD and magnetization cycles

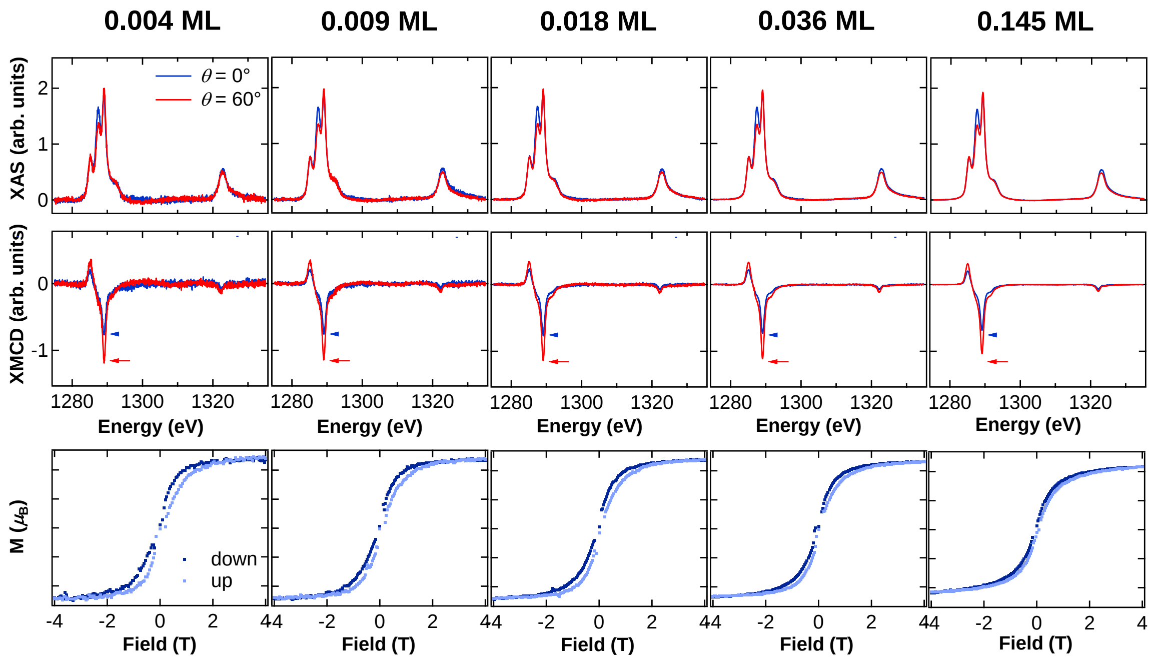

Figure S3: X-ray absorption spectra and magnetization curves of Dy on the Nb:STO(001) surface, for Dy coverages in the range between $\Theta_{\mathrm{Dy}} = 0.004\,\mathrm{ML}$ and $\Theta_{\mathrm{Dy}} = 0.145\,\mathrm{ML}$. The XAS and XMCD spectra are measured at the Dy $M_{4,5}$ edges at a temperature $T = 2.5\,\mathrm{K}$ and a magnetic field $B = 5\,\mathrm{T}$, both at normal ($\theta = 0\,°$) and at grazing incidence ($\theta = 60\,°$). All spectra at each coverage are normalized to the total integral of the XAS recorded at normal incidence. The red and blue arrows in each XMCD panel indicate the magnitude of the XMCD at the smallest coverage of $\Theta_{\mathrm{Dy}} = 0.004\,\mathrm{ML}$ for grazing and normal incidence, respectively. The magnetization curves are recorded at $T = 2.5\,\mathrm{K}$ and $\mathrm{d}B/\mathrm{d}t = 12.5\,\mathrm{mT/s}$, at grazing ($\theta = 60\,°$) incidence.

We have characterized the magnetic properties of Dy/Nb:STO(001) in a wide range of Dy surface concentrations. Figure S3 shows the XAS, XMCD and magnetization curves in the range of coverages from $\Theta_{\mathrm{Dy}} = 0.004\,\mathrm{ML}$ to $\Theta_{\mathrm{Dy}} = 0.145\,\mathrm{ML}$. Across the whole series, the XAS does not show any appreciable lineshape variation. The XMCD is barely affected by the increase of Dy concentration. It decreases by less than 4% for a coverage increase from 0.004 to 0.036 ML, corresponding to a reduction of Dy monomers from 98% to 86% for a statistical distribution of adatoms on a square lattice.[5] At a coverage $\Theta_{\mathrm{Dy}} = 0.145\,\mathrm{ML}$, where small clusters of a few atoms are formed, the XMCD is decreased by 10% with respect

to $\Theta_{Dy} = 0.004$ ML. Correspondingly, the opening of the magnetization cycles progressively reduces with increasing Dy coverage, suggesting that the magnetization of dimers and larger clusters is considerably less stable than that of Dy individual atoms on the $SrTiO_3(001)$ surface.

XAS, XMCD, XLD and magnetization cycles of Ho/Nb:STO(001)

Fig. S4a displays the $M_{4,5}$ XAS, XMCD, and XLD for Ho atoms on the Nb:STO(001) surface. As for Dy, we do not observe changes of the XAS and XMCD for Ho coverages up to $\Theta_{Ho} = 0.036$ ML. The spectral lineshape is typical for a 4f shell occupation $n = 10$. Thus, similar to the case of Dy, adsorption of Ho on the STO(001) surface leads to a decrease of one electron in the occupation of this shell, which is characterized by $n = 11$ for Ho atoms in the gas phase. The larger XMCD amplitude at grazing incidence than at normal incidence indicates an in-plane magnetic anisotropy, as found for Dy. This is confirmed by the spectral shape of the XLD at the M_5 edge. Contrary to the case of Dy, however, the magnetization cycles are paramagnetic at both normal and grazing incidence, for $T = 5$ K (see Fig. S4b), and they remain closed even at a lower temperature of $T = 2.5$ K, as shown in Fig. S4c.

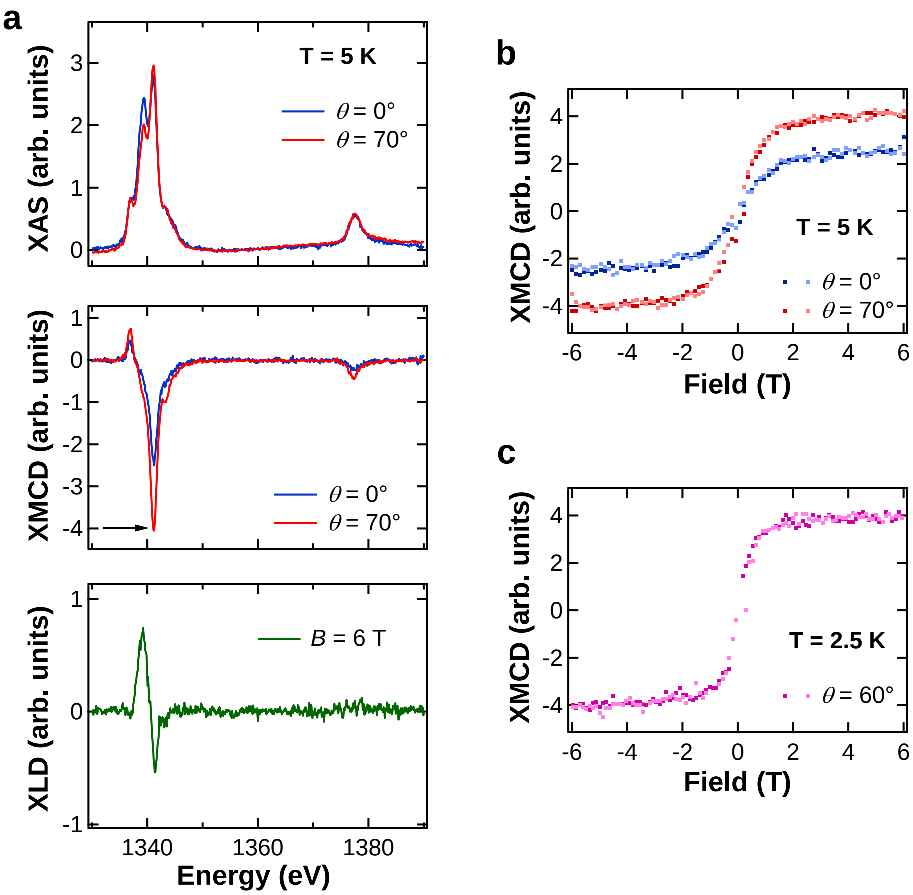

Figure S4: X-ray absorption spectra and magnetization curves of Ho individual atoms on a Nb:STO(001) surface, for a Ho coverage $\Theta_{\mathrm{Ho}} = 0.036$ ML. (a) XAS, XMCD and XLD spectra measured at the Ho $M_{4,5}$ edges and a temperature $T = 5$ K; the XAS and XMCD were recorded at a magnetic field $B = 6$ T; the XLD was recorded at grazing incidence ($\theta = 70\,^\circ$), for $B = 6$ T. (b) Magnetization curves recorded at $T = 5$ K and $\mathrm{d}B/\mathrm{d}t = 33.3$ mT/s, at normal ($\theta = 0\,^\circ$) and grazing ($\theta = 70\,^\circ$) incidence. The curves are normalized to the corresponding XMCD. Dark symbols are used for the downward magnetic field ramps (*i. e.* from positive to negative field), while light symbols are used for the upward field ramps. (c) Magnetization curves recorded at grazing incidence ($\theta = 60\,^\circ$) and $\mathrm{d}B/\mathrm{d}t = 33.3$ mT/s at $T = 2.5$ K and a Ho coverage $\Theta_{\mathrm{Ho}} = 0.018$ ML.

DFT calculations

Further details on the simulation cell and the methodology

In order to compare the properties of the different Dy adsorption sites on the two possible STO surface terminations, we considered an asymmetric slab, as depicted in Fig. 1d, placing

Dy ions either on top or below the slab. A 2×2 in-plane cell, in terms of the STO lattice constant (0.3905 nm), was used to simulate the properties of the isolated adatoms. The slab is composed of three and a half repetitions of the STO bulk cell in the direction perpendicular to the surface. For the analysis of the magnetic polarization of the substrate, a 4×4 in-plane cell was used, since the aim was to check the extension of the polarization given by a single Dy adatom. Vacuum space of 15 Å was considered in all the supercells in order to avoid slab replica spurious interactions. The RKmax parameter, that in the Wien2K code determines the size of the plane wave basis set, was chosen such as to ensure the number of APW's per atom ranged from 100 to 150 depending on the size of the simulation cell (equivalent in terms of cut-off energy to a 150-220 eV range). Concerning k-point integration in the Brillouin zone, we considered for the structural relaxation a 6×6 k-points grid for the 2×2 cell, and a 3×3 k-points grid for the 4×4 cell, shifted from Γ. A finer k mesh was used for the electronic properties (band structure, LDOS, orbital occupations). The convergence of the results as a function of the above parameters was checked. Moreover, local orbitals were added to the basis set, in order to describe more accurately s and p semi-core states within 80 eV from the Fermi level. For Dy, Ti, Sr and O atoms, muffin tin radii of 2.40, 1.74, 2.48, and 1.50 a.u. were used, respectively.

GGA+U vs. on-site B3LYP

We present here the results obtained by the DFT+U method in its Around Mean Field version,[6] and rotationally invariant implementation.[7] An on-site Hubbard correction term on the f orbital of Dy was added, choosing a value of $U = 7$ eV and $J = 0.82$ eV, as previously used for Dy adatoms on metal substrates.[8] Tests were carried out increasing the value of U up to 10 eV and only minor modifications were observed. In Table S1 we compare the results obtained by the GGA+U method with the ones obtained using the on-site B3LYP method already presented in the main text. The total energy of Dy at different adsorption sites is given, following the same definition as in Table 1 of the main text, together with

Table S1: Comparison between the GGA+U and on-site B3LYP calculations for the different adsorption sites at the two possible TiO_2 and SrO terminations. Total energy values (in eV) are referred to the most stable solution. In the case of the SrO termination, energies (in brackets) are referred to the most stable solution among the ones on the same termination. Electronic occupations of the 4f Dy adatom orbital are also reported.

		GGA+U		on-site B3LYP	
Term.	Ads. site	Energy (eV)	n (e^-)	Energy (eV)	n (e^-)
TiO_2	hollow	**+0.00**	9.12	**+0.00**	9.00
	top-O	+1.70	9.79	+2.61	9.39
	top-Ti	+2.29	9.82	+3.22	9.71
SrO	top-O	+1.68(**+0.00**)	9.80	+2.34(**+0.00**)	9.38
	bridge	+2.68(**+1.00**)	9.69	+2.95(**+0.61**)	9.04
	top-Sr	+4.55(+2.87)	9.77	+4.87(+2.53)	9.37

the occupation of the Dy 4f orbitals. We observe that the GGA+U method predicts the same trend as the on-site B3LYP for the relative stability of the different adsorption sites, although some variations are found in the absolute values. As anticipated in the main text, the on-site B3LYP method leads to results which are generally in better agreement with the experimental observation of a $4f^9$ configuration for the Dy 4f shell. An occupation closer to $4f^{10}$ is always associated with a reduction of the occupation of the 6s, 6p and 5d orbitals composing the valence shell, leading to a different distribution of the electrons within the spdf shell, while charge transfer to the surface is similar for both valences.

Spin and orbital projected band structures: 2DEG at the STO surface

As discussed in the main text, the presence of Dy adatoms on both TiO_2 and SrO terminations of the STO(001) surface induces a metallization of the substrate and the appearance of parabolic conduction bands with Ti-d_{xy} character as depicted in Fig. 1g of the main text. In Fig. S5 we present the depth dependence of such bands for the TiO_2/hollow (panels from a to d) and SrO/top-O adsorption sites (panels e and f), in the case of the majority spin

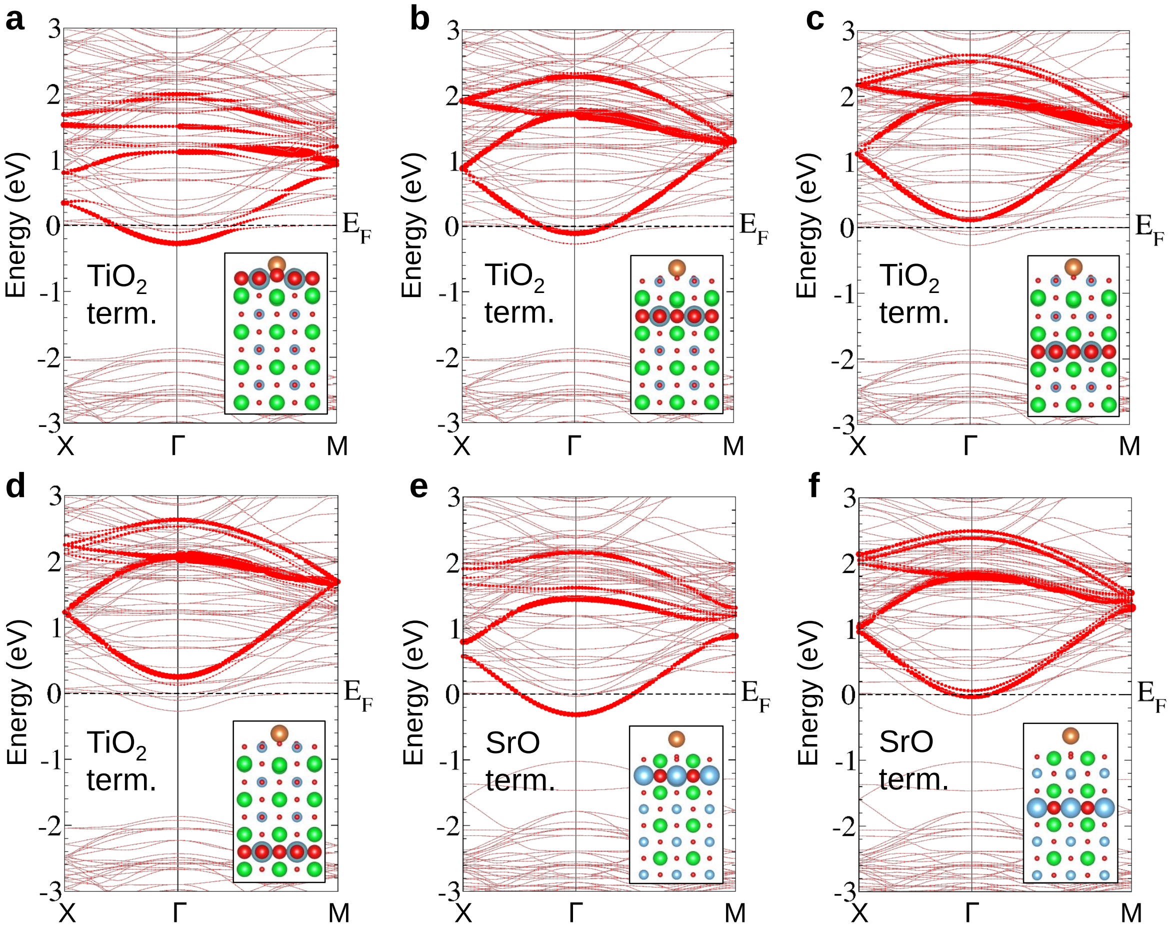

Figure S5: Majority spin (↑) orbitally-projected electronic band structure in the [−3 eV,+3 eV] energy range around the Fermi level. The size of the red circles is proportional to the Ti-d_{xy} orbital contribution in the case of a Dy adatom (a-d) at the hollow site on the TiO_2 terminated surface, and (e,f) for a Dy adatom at the top-O position on the SrO terminated surface. The contribution of Ti atoms at increasing depth within the substrate is shown moving from (a) to (d) and from (e) to (f), as evidenced by the structural sketches in the insets of the various panels.

band structure. We note that the metallic states are mainly localized in the surface or subsurface TiO_2 planes, similarly to what was previously observed by DFT calculations for the 2DEG states induced by intrinsic O-vacancies in STO interfaces and surfaces.[9,10] Moreover the predominant Ti-d_{xy} character of the metallic bands is observed independently of the termination on top of which the Dy adatoms are adsorbed. This means that the 2DEG bands will exist on the entire STO(001) surface, not just at islands of a particular termination.

Details about the atomic multiplet calculations

Point charges and crystal field parameters

Table S2: Point charges used to calculate the crystal field parameters at the different adsorption sites (hollow, top-O and top-Ti) of the TiO_2 termination. The Dy atom position is the origin of the coordinate system.

TiO_2/hollow

q (e)	x (Å)	y (Å)	z (Å)
−0.007	0.000	0.000	0.700
−1.285	1.891	0.000	−1.126
−1.285	−1.891	0.000	−1.126
−1.285	0.000	1.891	−1.126
−1.285	0.000	−1.891	−1.126
2.092	1.953	1.953	−1.552
2.092	−1.953	1.953	−1.552
2.092	1.953	−1.953	−1.552
2.092	−1.953	−1.953	−1.552
1.544	0.000	0.000	−3.695
−1.264	1.953	1.953	−3.381
−1.264	−1.953	1.953	−3.381
−1.264	1.953	−1.953	−3.381
−1.264	−1.953	−1.953	−3.381
1.585	−3.905	−0.000	−3.481
1.585	3.905	−0.000	−3.481
1.585	−0.000	−3.905	−3.481
1.585	0.000	3.905	−3.481

TiO_2/top-O

q (e)	x (Å)	y (Å)	z (Å)
−1.301	0.000	0.000	−2.121
2.112	1.953	0.000	−2.617
2.112	−1.953	0.000	−2.617
−1.228	1.953	−1.953	−2.521
−1.228	−1.953	−1.953	−2.521
−1.228	1.953	1.953	−2.521
−1.228	−1.953	1.953	−2.521
−1.226	−0.000	−3.905	−2.507
−1.226	0.000	3.905	−2.507
−1.191	−3.905	−0.000	−2.528
−1.191	3.905	−0.000	−2.528

TiO_2/top-Ti

q (e)	x (Å)	y (Å)	z (Å)
2.103	0.000	0.000	−3.351
−1.233	0.000	−1.953	−3.211
−1.233	0.000	1.953	−3.211
−1.233	−1.953	0.000	−3.211
−1.233	1.953	0.000	−3.211
2.194	−3.905	0.000	−3.355
2.194	3.905	0.000	−3.355
2.194	0.000	−3.905	−3.355
2.194	0.000	3.905	−3.355

The crystal field parameters used in atomic multiplet calculations for the 4f orbitals were obtained by using a point charge model based, for each adsorption site, on the DFT optimized adsorption geometry and the corresponding Bader charges of the ligands surrounding the

Table S3: Point charges used to calculate the crystal field parameters at the different adsorption sites (top-O, bridge and top-Sr) of the SrO_2 termination. The Dy atom position is the origin of the coordinate system.

SrO/bridge

q (e)	x (Å)	y (Å)	z (Å)
−1.427	0.000	1.599	−1.531
−1.427	0.000	−1.599	−1.531
1.479	−2.096	0.000	−2.284
1.479	2.096	0.000	−2.284
−1.270	0.000	0.000	−3.996
2.110	−0.000	1.953	−4.034
2.110	−0.000	−1.953	−4.034
−1.294	−1.953	1.953	−3.927
−1.294	1.953	−1.953	−3.927
−1.294	−1.953	−1.953	−3.927
−1.294	1.953	1.953	−3.927
−1.333	−3.905	1.953	−2.023
−1.333	3.905	1.953	−2.023
−1.333	−3.905	−1.953	−2.023
−1.333	3.905	−1.953	−2.023
1.583	−1.953	−3.905	−2.119
1.583	−1.953	3.905	−2.119
1.583	1.953	−3.905	−2.119
1.583	1.953	3.905	−2.119

SrO/top-O

q (e)	x (Å)	y (Å)	z (Å)
−1.435	−0.000	−0.000	−2.088
1.541	−1.953	−1.953	−2.611
1.541	1.953	1.953	−2.611
1.541	1.953	−1.953	−2.611
1.541	−1.953	1.953	−2.611
−1.322	−3.905	−0.000	−2.427
−1.322	3.905	−0.000	−2.427
−1.322	−0.000	−3.905	−2.427
−1.322	0.000	3.905	−2.427

SrO/top-Sr

q (e)	x (Å)	y (Å)	z (Å)
1.531	0.000	0.000	−4.185
−1.346	1.953	1.953	−3.964
−1.346	−1.953	1.953	−3.964
−1.346	1.953	−1.953	−3.964
−1.346	−1.953	−1.953	−3.964
1.569	−3.905	0.000	−4.169
1.569	3.905	0.000	−4.169
1.569	−0.000	−3.905	−4.169
1.569	0.000	3.905	−4.169
−1.282	1.953	0.000	−5.941
−1.282	−1.953	0.000	−5.941
−1.282	0.000	1.953	−5.941
−1.282	0.000	−1.953	−5.941
2.190	1.953	1.953	−5.898
2.190	−1.953	1.953	−5.898
2.190	1.953	−1.953	−5.898
2.190	−1.953	−1.953	−5.898

Table S4: Rescaled (see text) crystal field Wybourne parameters (in units of meV) for the six Dy adsorption sites on the two surface terminations of STO(001).

**Termination/site**	A_2^0	A_2^2	A_4^0	A_4^2	A_4^4	A_6^0	A_6^2	A_6^4	A_6^6
TiO_2/hollow	−36.3	–	75.9	–	171.9	691.7	–	62.5	–
TiO_2/top-O	420.3	−120.4	140.7	−27.1	−5.9	54.8	−2.9	−3.5	−0.2
TiO_2/top-Ti	−41.8	–	−19.9	–	−0.8	−4.8	–	0.3	–
SrO/top-O	577.6	–	185.3	–	8.8	64.1	–	2.0	–
SrO/bridge	162.8	−335.8	−86.7	−144.8	30.6	−5.7	−0.2	30.8	−6.4
SrO/top-Sr	−52.8	–	−4.9	–	−1.0	−0.7	–	−0.2	–

Dy atoms. In the case of Dy on the TiO_2/hollow site, we have explicitly included a small negative charge above the Dy atom, in order to reproduce the anisotropic charge density associated with the Dy spd electrons. Tables S2 and S3 show the point charges used in the case of the TiO_2 and the SrO terminations, respectively. With these, we calculated the crystal field parameters, in Wybourne notation, using the "pointc" module[11] within the software McPhase.[12] In order to compensate for the well-known underestimation of the crystal field parameters of order 4 and 6 with respect to those of order 2 by the point charge model, we have rescaled the crystal field by factors κ_l ($l = 2, 4, 6$) following the procedure outlined by Singha *et al.*[13] ($\kappa_2 = \sqrt{2}$, $\kappa_4 = 2\sqrt{2}$, $\kappa_6 = 4$), thus obtaining the modified Wybourne parameters used in Quanty for the atomic multiplet calculations, indicated as A_l^m in Table S4 (with $m = 0, 2, 4, 6$).

Simulated XAS, XMCD and magnetization cycles for each adsorption site

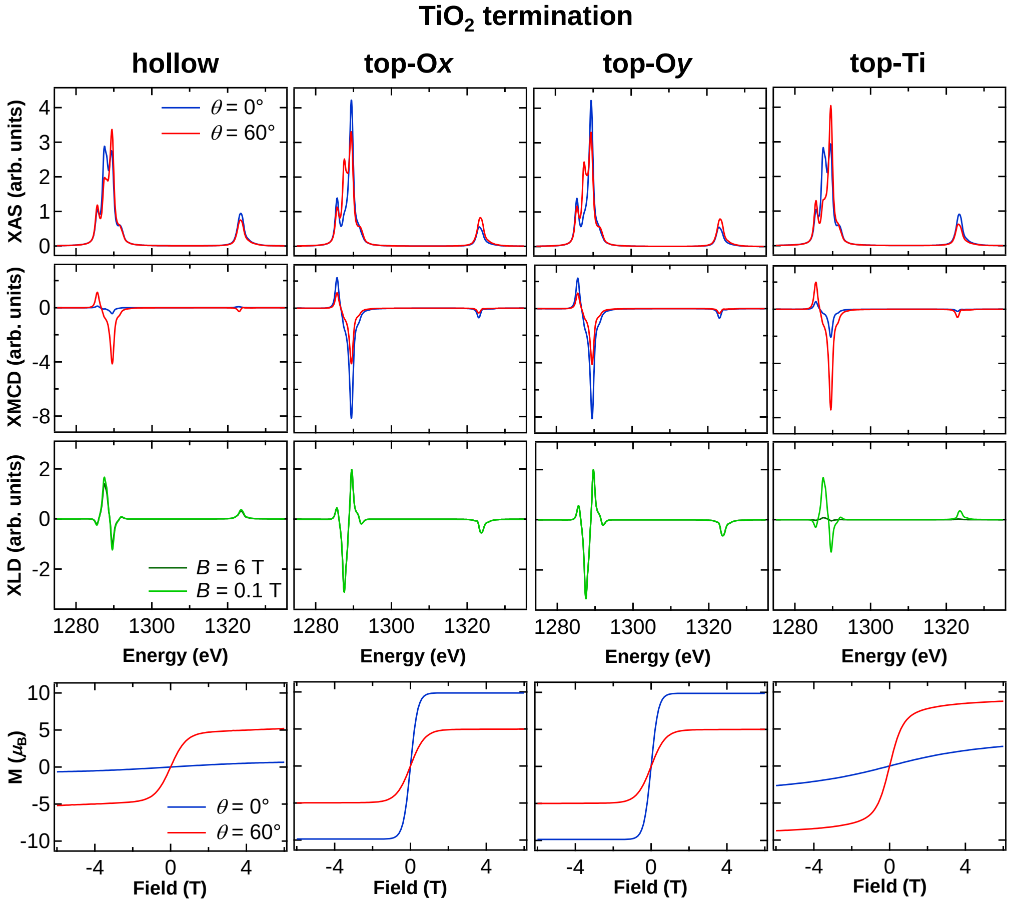

Figure S6: XAS and XMCD spectra at the Dy $M_{4,5}$ edges and magnetization loops simulated by means of atomic multiplet calculations for each Dy adsorption site on the TiO_2 termination of the STO(001) surface. The simulations are performed for a temperature $T = 2.5\,\mathrm{K}$. A magnetic field $B = 5\,\mathrm{T}$ is applied at $\theta = 0^\circ$ and $\theta = 60^\circ$ for the XAS and XMCD, while the XLD was calculated for $\theta = 60^\circ$.

Figures S6 and S7 show the XAS and XMCD spectra calculated by atomic multiplet calculations at the Dy $M_{4,5}$ edges for each adsorption site at the TiO_2 and SrO terminations, respectively. The simulations are for a temperature $T = 2.5\,\mathrm{K}$ and a magnetic field $B = 5\,\mathrm{T}$. The bottom graphs in each column show the corresponding magnetization cycle at thermodynamical equilibrium. The magnetic field and the X-ray beam rotate in the xz-plane when

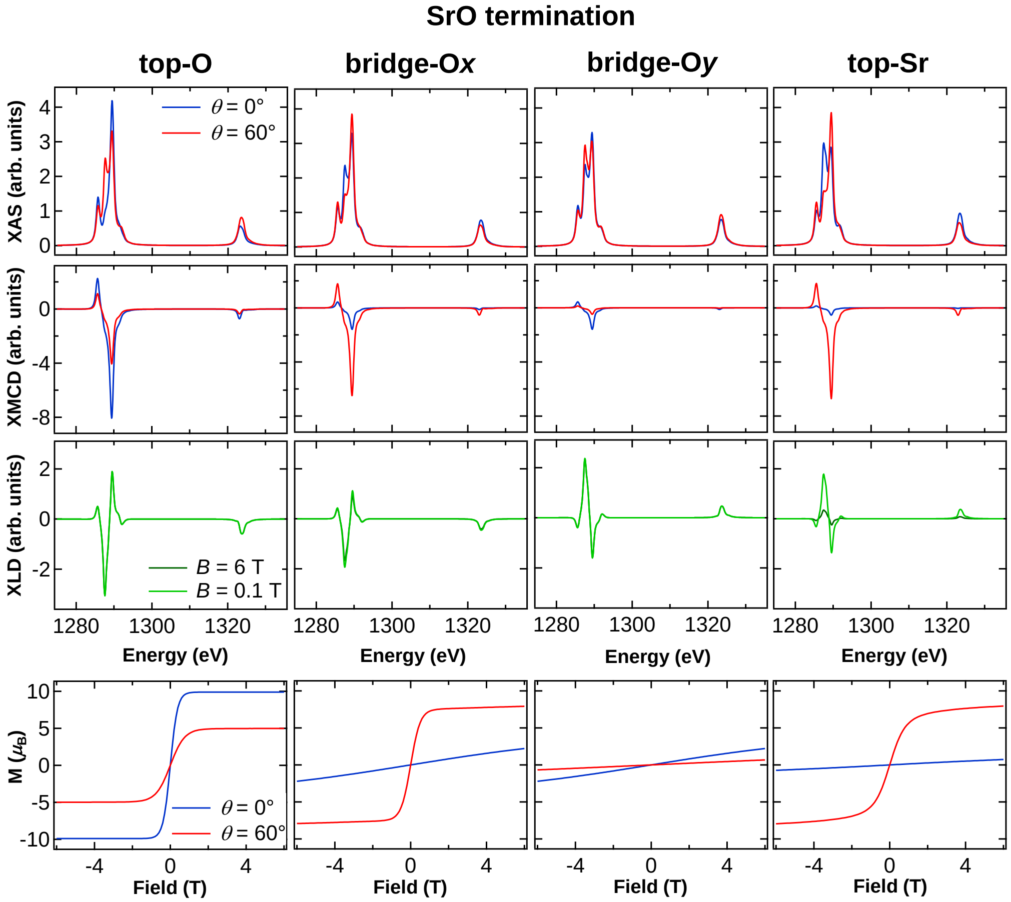

Figure S7: XAS and XMCD spectra at the Dy $M_{4,5}$ edges and magnetization loops simulated by means of atomic multiplet calculations for each Dy adsorption site on the SrO termination of the STO(001) surface. The simulations are performed for a temperature $T = 2.5\,\mathrm{K}$. A magnetic field $B = 5\,\mathrm{T}$ is applied at $\theta = 0\,°$ and $\theta = 60\,°$ for the XAS and XMCD, while the XLD was calculated for $\theta = 60\,°$.

going from normal ($\theta = 0\,°$) to grazing ($\theta = 60\,°$) incidence. The two-fold symmetry of the top-O site on the TiO_2 termination and of the bridge site on the SrO termination leads to the coexistence of two Dy species on each site. In fact, the two Ti next-nearest neighbours for the top-O site, and the two O nearest neighbours for the bridge site may either be aligned along the x direction (sites indicated as "top-Ox" and "bridge-Ox") or along the y direction (sites indicated as "top-Oy" and "bridge-Oy"). While in the top-O case both orientations correspond to a magnetic hard axis, for the bridge site the "bridge-Ox", with the Dy magnetic moments aligned towards the neighbour O atoms, is the magnetic easy axis. Among

the other sites, all showing four-fold symmetry, the hollow and top-Ti on the TiO_2 termination and the top-Sr on the SrO termination exhibit in-plane magnetic anisotropy, while the top-O site on the SrO termination has a strong out-of-plane anisotropy.

Details about the calculation of the magnetization relaxation time τ

Theoretical model

The spin-phonon scattering probability between an initial $|i\rangle$ and a final $|j\rangle$ eigenstate of the Dy/STO(001) atomic system, following the model proposed by Fort *et al.*,[14] can be written as:

$$G_{\mathrm{ph}}^{ij} = \begin{cases} C_{\mathrm{ph}}\, M_{\mathrm{ph}}^{ij}\, |\Delta E_{ij}|^3\, n_{ij} & \text{for} \quad \Delta E_{ij} < 0 \quad \text{(phonon absorption)} \\ \\ C_{\mathrm{ph}}\, M_{\mathrm{ph}}^{ij}\, |\Delta E_{ij}|^3\, (n_{ij}+1) & \text{for} \quad \Delta E_{ij} > 0 \quad \text{(phonon emission)} \end{cases}, \tag{S1}$$

where C_{ph} is a constant characteristic for the spin-phonon scattering cross-section in Dy/STO(001), $\Delta E_{ij} = E_i - E_j$ is the difference of the energies of eigenstates $|i\rangle$ and $|j\rangle$, $n_{ij} = \{\exp[|\Delta E_{ij}|/(k_{\mathrm{B}}T)] - 1\}^{-1}$ is the Bose-Einstein distribution of phonon population, k_{B} is Boltzmann's constant, and T is the temperature. The spin-phonon scattering matrix elements, following the notation by Cervetti *et al.*,[15] are defined as:

$$M_{\mathrm{ph}}^{ij} = |\langle j|J_-^2|i\rangle|^2 + |\langle j|J_+^2|i\rangle|^2 + 2|\langle j|\{J_-, J_z\}|i\rangle|^2 + 2|\langle j|\{J_+, J_z\}|i\rangle|^2, \tag{S2}$$

with $J_\pm = J_x \pm iJ_y$. The same matrix elements were previously used for Dy based molecular complexes.[16]

Analogously, following Delgado and Fernández-Rossier,[17] we write the spin-electron scat-

tering probability as:

$$G_{\text{el}}^{ij} = C_{\text{el}}\, M_{\text{el}}^{ij}\, \frac{\Delta E_{ij}}{e^{\frac{\Delta E_{ij}}{k_{\text{B}}T}} - 1}, \tag{S3}$$

where C_{el} is a constant characteristic for the spin-electron scattering cross-section in Dy/STO(001), which in turn depends on the (unknown) electronic density of the STO(001) substrate, and the spin-electron scattering matrix elements are defined as:

$$M_{\text{el}}^{ij} = |\langle j|J_{-}|i\rangle|^2 + |\langle j|J_{+}|i\rangle|^2 + 2|\langle j|J_z|i\rangle|^2, \tag{S4}$$

which assumes that the exchange of the Dy 4f shell with the conduction-electron spins $\mathbf{s}$ is accurately approximated by the bilinear Heisenberg exchange in the form $\mathbf{J}\cdot\mathbf{s}$, despite some deviations and/or extra terms due to the orbital component of the total moment $\mathbf{J}$ are to be expected.[18]

With the knowledge of G_{ph}^{ij} and G_{el}^{ij}, we can solve the set of N_{lev} differential rate equations (N_{lev} is the total number of eigenstates of the Dy/STO(001) system), relating the population $P_i(t)$ of state $|i\rangle$ at time t with the probability of phonon or electron related scattering to/from the other states $|j\rangle$, with $j \neq i$:

$$\frac{\partial P_i(t)}{\partial t} = -P_i(t) \sum_{\substack{j=1 \\ j\neq i}}^{N_{\text{lev}}} \left(G_{\text{ph}}^{ij} + G_{\text{el}}^{ij}\right) + \left[\sum_{\substack{j=1 \\ j\neq i}}^{N_{\text{lev}}} P_j(t)\left(G_{\text{ph}}^{ji} + G_{\text{el}}^{ji}\right)\right], \tag{S5}$$

with the boundary conditions $P_1(0) = 1$ (where $|1\rangle$ is the ground-state of the system) and $P_j(0) = 0$ for $j = 2\ldots N_{\text{lev}}$. The time evolution of the population of the ground state $|1\rangle$, $P_1(t)$, is then fitted with an exponential decay function of the type:

$$P_1(t) = P_1(0)e^{-\frac{t}{\tau}},$$

allowing us to determine τ.

In the case of the Dy^{hollow} species, the magnetic field dependence of τ shown in Fig. 5b

of the main text is calculated based on the values $C_{\mathrm{ph}} = 2.1 \times 10^{-2}$ and $C_{\mathrm{el}} = 7.5 \times 10^{-5}$.

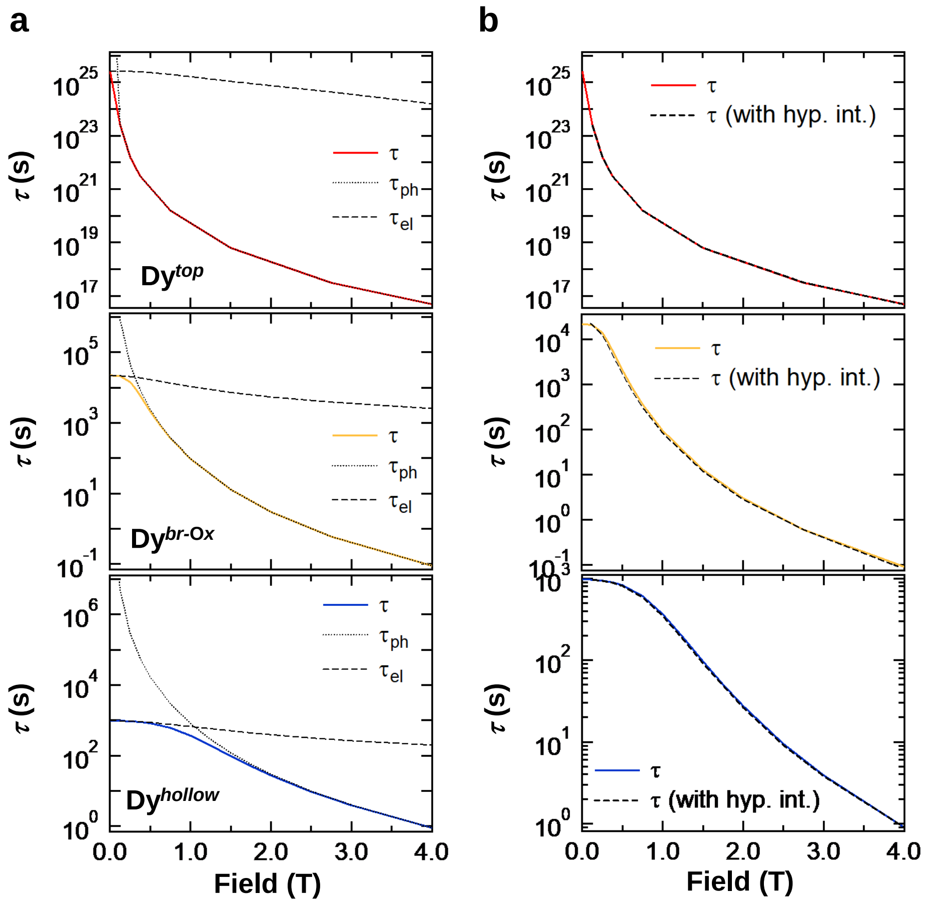

Figure S8: (a) Intrinsic magnetization relaxation time τ and its decomposition into a spin-phonon contribution (τ_{ph}) and a spin-electron contribution (τ_{el}) at $T = 2.5\,\mathrm{K}$ and $\theta = 60\,°$; (b) Comparison between the magnetic field dependence of τ calculating with and without including the coupling of the $4f$ magnetic moment with the nuclear spin $I = 5/2$.

Fig. S8a shows the decomposition of the intrinsic magnetization relaxation time τ into a spin-phonon contribution (τ_{ph}) and a spin-electron contribution (τ_{el}). The values for τ are those displayed in Fig. 5b of the main text. It is evident that for all three adsorption sites the calculated values of τ are dominated by the spin-electron scattering at low magnetic fields, while the spin-phonon scattering contribution prevails at high magnetic field. In Fig. S8b we compare the magnetic field dependence of τ as calculated including or neglecting the coupling of the 4f magnetic moment with the nuclear spin I. Such coupling is effective

only for Dy isotopes with $I \neq 0$: ^{161}Dy with relative abundance of 18.9% and ^{163}Dy with relative abundance of 24.9%, both having $I = 5/2$. As evident from the figure, there is negligible difference between the two cases in the magnetic field range of our experimental investigations of τ ($B > 0.1\,$T).

Estimate of the secondary electrons contribution to the value of the relaxation time

The X-ray flux dependent contribution $\tau_{\rm sec}(\Phi)$ of secondary electrons to the measured value of the relaxation time $\tau_{\rm exp}(\Phi)$ can be estimated by noting that the latter may be expressed as $\tau_{\rm exp}^{-1}(\Phi) = \tau^{-1} + \tau_{\rm sec}^{-1}(\Phi)$, where τ is the intrinsic relaxation time of the Dy magnetization and Φ is the X-ray flux. Because of the limited magnetic field range (between 0.1 and 1 T) studied in our investigations of τ, we assume that $\tau_{\rm sec}(\Phi)$ does not depend on magnetic field, and we determine its X-ray flux dependence at $B = 0.375\,$T. By measuring $\tau_{\rm exp}$ at two X-ray fluxes $\Phi_{\rm lf}$ and $\Phi_{\rm hf} = 4 \times \Phi_{\rm lf}$ (where the indices "lf" and "hf" stand for "low flux" and "high flux", respectively), we obtain a system of two equations:

$$\begin{cases} \tau_{\rm exp}^{-1}(\Phi_{\rm lf}) = \tau^{-1} + \tau_{\rm sec}^{-1}(\Phi_{\rm lf}) \\ \\ \tau_{\rm exp}^{-1}(\Phi_{\rm hf}) = \tau^{-1} + \tau_{\rm sec}^{-1}(\Phi_{\rm hf}) \end{cases} . \qquad \text{(S6)}$$

By combining the two equations above, we obtain:

$$\tau_{\rm sec}(\Phi_{\rm lf}) = \frac{a\tau_{\rm sec}(\Phi_{\rm hf})}{a - \tau_{\rm sec}(\Phi_{\rm hf})},$$

where $a = \frac{\tau_{\rm exp}(\Phi_{\rm lf})\tau_{\rm exp}(\Phi_{\rm hf})}{\tau_{\rm exp}(\Phi_{\rm lf}) - \tau_{\rm exp}(\Phi_{\rm hf})}$. The obvious condition $\tau_{\rm sec}(\Phi_{\rm lf}) \geq 0$ imposes that $\tau_{\rm sec}(\Phi_{\rm hf}) \leq a$, setting an upper limit for the secondary electrons contribution to the relaxation time in our "high flux" conditions, which correspond to the X-ray flux used for recording all the magnetization cycles presented in this manuscript. At $B = 0.375$T, where we record $\tau_{\rm exp}(\Phi_{\rm lf}) = 800 \pm 200\,$s

and $\tau_{\rm exp}(\Phi_{\rm hf}) = 160 \pm 20\,{\rm s}$, we then obtain $\tau_{\rm sec}(\Phi_{\rm hf}) \leq 200 \pm 40\,{\rm s}$. The lower limit for $\tau_{\rm sec}(\Phi_{\rm hf})$, on the other hand, is represented by the largest value of $\tau_{\rm exp}(\Phi_{\rm hf})$ in our magnetic-field dependent series, which is $190 \pm 30\,{\rm s}$ at $B = 0.1\,{\rm T}$. Due to the large uncertainty in these values, we take $\tau_{\rm sec}(\Phi_{\rm hf})$ to lie in the middle of its allowed range, corresponding to $\tau_{\rm sec}(\Phi_{\rm hf}) = 195 \pm 35\,{\rm s}$. With this value, we estimate the intrinsic value of the magnetization relaxation time at $B = 0.375\,{\rm T}$ by using the bottom equation in (S6), giving $\tau = 900 \pm 40\,{\rm s}$. This, in turn, allows us to estimate the secondary electrons contribution to the relaxation time in our "low flux" conditions (corresponding to the X-ray flux used for recording the values of $\tau_{\rm exp}$ shown in Fig. 5a of the main text), by using the top equation in (S6), resulting in $\tau_{\rm sec}(\Phi_{\rm lf}) = 7100 \pm 200\,{\rm s}$.

Taking into account the above estimated values of $\tau_{\rm sec}$, with the condition $\tau_{\rm sec}^{-1}(\Phi = 0) = 0$, we find a quadratic dependence of the secondary electrons contribution to the relaxation time on the X-ray flux:

$$\tau_{\rm sec}^{-1}(\Phi) = b \left(\frac{\Phi}{\Phi_{\rm hf}} \right)^2 , \qquad \text{(S7)}$$

with $b = (5 \pm 1) \times 10^{-3}\,{\rm s}^{-1}$.

References

1. Biswas, A.; Rossen, P. B.; Yang, C.-H.; Siemons, W.; Jung, M.-H.; Yang, I. K.; Ramesh, R.; Jeong, Y. H. Universal Ti-Rich Termination of Atomically Flat $SrTiO_3$ (001), (110), and (111) Surfaces. *Applied Physics Letters* **2011**, *98*, 051904.

2. Johnston, K.; Castell, M. R.; Paxton, A. T.; Finnis, M. W. $SrTiO_3$(001)(2×1) Reconstructions: First-Principles Calculations of Surface Energy and Atomic Structure Compared with Scanning Tunneling Microscopy Images. *Phys. Rev. B* **2004**, *70*, 085415.

3. Jiang, Q. D.; Zegenhagen, J. c(6×2) and c(4×2) Reconstruction of $SrTiO_3$(001). *Surface Science* **1999**, *425*, 343–354.

4. Lanier, C. H.; van de Walle, A.; Erdman, N.; Landree, E.; Warschkow, O.; Kazimirov, A.; Poeppelmeier, K. R.; Zegenhagen, J.; Asta, M.; Marks, L. D. Atomic-Scale Structure of the $SrTiO_3$(001) c(6×2) Reconstruction: Experiments and First-Principles Calculations. *Phys. Rev. B* **2007**, *76*, 045421.

5. Sykes, M. F.; Glen, M. Percolation Processes in Two Dimensions. I. Low-Density Series Expansions. *Journal of Physics A: Mathematical and General* **1976**, *9*, 87–95.

6. Czyżyk, M. T.; Sawatzky, G. A. Local-Density Functional and On-Site Correlations: The Electronic Structure of La_2CuO_4 and $LaCuO_3$. *Phys. Rev. B* **1994**, *49*, 14211–14228.

7. Liechtenstein, A. I.; Anisimov, V. I.; Zaanen, J. Density-Functional Theory and Strong Interactions: Orbital Ordering in Mott-Hubbard Insulators. *Phys. Rev. B* **1995**, *52*, R5467–R5470.

8. Shick, A.; Denisov, A. Magnetism of 4f-Atoms Adsorbed on Metal and Graphene Substrates. *Journal of Magnetism and Magnetic Materials* **2019**, *475*, 211–215.

9. Shen, J.; Lee, H.; Valentí, R.; Jeschke, H. O. Ab Initio Study of the Two-Dimensional

Metallic State at the Surface of $SrTiO_3$: Importance of Oxygen Vacancies. *Phys. Rev. B* **2012**, *86*, 195119.

10. Gao, L.; Demkov, A. A. Spin-Polarized Two-Dimensional t_2g Electron Gas: Ab Initio Study of EuO Interface with Oxygen-Deficient $SrTiO_3$. *Phys. Rev. B* **2018**, *97*, 125305.

11. Bauer, E.; Rotter, M. *Properties and Applications of Complex Intermetallics*; Book Series on Complex Metallic Alloys Volume 2; WORLD SCIENTIFIC, 2009; Vol. Volume 2; pp 183–248.

12. Rotter, M. Using McPhase to Calculate Magnetic Phase Diagrams of Rare Earth Compounds. *Proceedings of the International Conference on Magnetism (ICM 2003)* **2004**, *272-276*, E481–E482.

13. Singha, A.; Sostina, D.; Wolf, C.; Ahmed, S. L.; Krylov, D.; Colazzo, L.; Gargiani, P.; Agrestini, S.; Noh, W.-S.; Park, J.-H.; Pivetta, M.; Rusponi, S.; Brune, H.; Heinrich, A. J.; Barla, A.; Donati, F. Mapping Orbital-Resolved Magnetism in Single Lanthanide Atoms. *ACS Nano* **2021**, *15*, 16162–16171.

14. Fort, A.; Rettori, A.; Villain, J.; Gatteschi, D.; Sessoli, R. Mixed Quantum-Thermal Relaxation in Mn_{12} Acetate Molecules. *Phys. Rev. Lett.* **1998**, *80*, 612–615.

15. Cervetti, C.; Rettori, A.; Pini, M. G.; Cornia, A.; Repollés, A.; Luis, F.; Dressel, M.; Rauschenbach, S.; Kern, K.; Burghard, M.; Bogani, L. The Classical and Quantum Dynamics of Molecular Spins on Graphene. *Nature Materials* **2016**, *15*, 164–168.

16. Lucaccini, E.; Briganti, M.; Perfetti, M.; Vendier, L.; Costes, J.-P.; Totti, F.; Sessoli, R.; Sorace, L. Relaxation Dynamics and Magnetic Anisotropy in a Low-Symmetry DyIII Complex. *Chemistry – A European Journal* **2016**, *22*, 5552–5562.

17. Delgado, F.; Fernández-Rossier, J. Spin Dynamics of Current-Driven Single Magnetic Adatoms and Molecules. *Phys. Rev. B* **2010**, *82*, 134414.

18. Coqblin, B.; Schrieffer, J. R. Exchange Interaction in Alloys with Cerium Impurities. *Phys. Rev.* **1969**, *185*, 847–853.
